# A practical and safe alternative method for skeletal cleaning for museum specimens using superworms (*Zophobas morio*)

**DOI:** 10.1371/journal.pone.0349669

**Published:** 2026-07-01

**Authors:** Fatemeh Rastekar, Niloofar Alaei Kakhki, Mansour Aliabadian, Morteza Monfared

**Affiliations:** 1 Department of Biology, Faculty of Science, Ferdowsi University of Mashhad, Mashhad, Iran; 2 Department of Biodiversity Monitoring, State Museum of Natural History Stuttgart, Stuttgart, Germany; 3 Research Department of Zoological Innovations, Institute of Applied Zoology, Faculty of Science, Ferdowsi University of Mashhad, Mashhad, Iran; Universidade Federal de Minas Gerais, BRAZIL

## Abstract

Clean and undamaged skeletons that maintain natural anatomical shape are essential for anatomical collections and natural history museums. Conventional methods, such as maceration, chemical treatments, or dermestid beetle colonies, although commonly used, often require long processing times, pose biohazard risks, and may damage delicate bones. This study explores the use of superworms (*Zophobas morio*) as a biological alternative for skeletal cleaning. Controlled cleaning trials were conducted using specimens from various vertebrate groups and a range of size classes, categorized as small, medium, and large after removal of superficial tissues. As the larva to specimen ratio strongly influences cleaning time and effectiveness, we first standardized our setup by processing all specimens in identical containers, each with approximately 700 grams of superworms, allowing us to assess the optimal ratio. Our results showed that a high larva to specimen ratio led to damage of fragile bones, while lower ratios resulted in increased cleaning times. Through multiple trials, we suggest that a larva to specimen ratio of 10–15 balances efficient cleaning with minimal risk of bone damage. Applying this ratio to additional bird skulls resulted in thorough cleaning with no observed bone damage. Superworms removed soft tissues within hours to days, depending on specimen size, and were able to clean internal cavities that are typically difficult to reach. Unlike dermestid beetle colonies, which include multiple life stages and pose a higher risk of infestation or egg dispersal, superworms are limited to the larval stage, reducing such risks. Our findings demonstrate that superworms offer a rapid, adaptable, and museum-safe alternative to conventional skeletal cleaning methods, providing an efficient and practical option for scientific and curatorial settings. Superworms are readily available from commercial breeders, and maintaining a colony is straightforward, further supporting their use as a viable alternative for skeletal preparation.

## Introduction

Skeletal specimens are essential tools in museums, for teaching, public display, and comparative scientific study [1,2]. Precise skeletal preparation ensures the preservation of complex anatomical features, facilitating detailed analysis and meaningful interpretation [3]. Museum osteological series provide foundational material for comparative anatomy and evolutionary studies, enabling researchers to trace functional adaptations and phylogenetic relationships across vertebrate taxa [4]. Advances in imaging techniques such as computed tomography (CT) scanning have further enhanced the ability to examine skeletal morphology in exquisite detail, facilitating analyses of convergent evolution and developmental patterns without damaging valuable specimens [5]. Recent studies on dippers and frogs illustrate how detailed skeletal analyses provide valuable insights into convergent evolution and developmental biology in vertebrates [6,7]. These findings underscore the importance of well-prepared skeletal specimens for uncovering evolutionary patterns that may not be evident from external features alone.

Preserving the structural integrity of the skeletal specimens requires the use of effective cleaning techniques. Poor preparation can cause irreversible damage, diminishing both the scientific value and the utility for public display [8]. Traditional skeletal preparation methods, such as burial, enzymatic digestion, and chemical treatment, have been employed in museums and scientific collections for decades [3]. While these approaches effectively remove soft tissues, they often pose significant drawbacks, including potential damage to fragile bone, long processing times, high operational costs, and environmental hazards related to chemical use [3,9,10].

Recently, biological cleaning using dermestid beetles (*Dermestes*) has become the preferred method for skeleton preparation in many leading natural history museums, such as the Natural History Museum in London [11], the Smithsonian National Museum of Natural History [12], and the Burke Museum of Natural History and Culture [13]. Dermestid beetles efficiently remove soft tissues while preserving the delicate bone structures, making this method a widely accepted alternative to traditional chemical and mechanical cleaning techniques [14]. However, despite its effectiveness and minimal bone damage, the method carries risks such as beetle escape and the potential for undetected egg deposition, all of which could threaten museum collections [14–19].

To address these limitations of the existing methods, this study investigates the use of superworms (*Zophobas morio*) as a safe and effective biological alternative to both traditional and dermestid-based skeletal cleaning techniques. Unlike dermestid beetles, which require complex containment and involve all life stages within the colony [14], superworm cleaning relies solely on the larval stage [20]. This ensures that all individuals actively contribute to tissue removal while eliminating risks related to adult emergence, reproduction, and unnoticed egg laying. Moreover, superworms do not pupate under crowded conditions [20], simplifying colony management and significantly reducing the chance of escapes or contamination in museum environments. Superworms undergo multiple molts before pupation, with optimal growth occurring at temperatures between 25–28°C and relative humidity levels of 60–70% [20]. Moreover, superworms are widely used in the pet and animal feed industries due to their high protein content and ease of rearing [20,21]. Together, these traits make superworms an accessible and efficient biological agent for skeletal cleaning, ideal for institutions seeking reliable alternatives. This new approach addresses the drawbacks of traditional and dermestid-based skeletal cleaning methods while offering key advantages, such as reducing risk of contamination in museums, minimizing damage to bones, simplifying colony management, and providing fast processing times. Ultimately, superworm-based cleaning presents a reliable, sustainable, and practical alternative with the potential to transform skeletal preparation practices in museums and research institutions.

## Materials and methods

**Fig 1:**
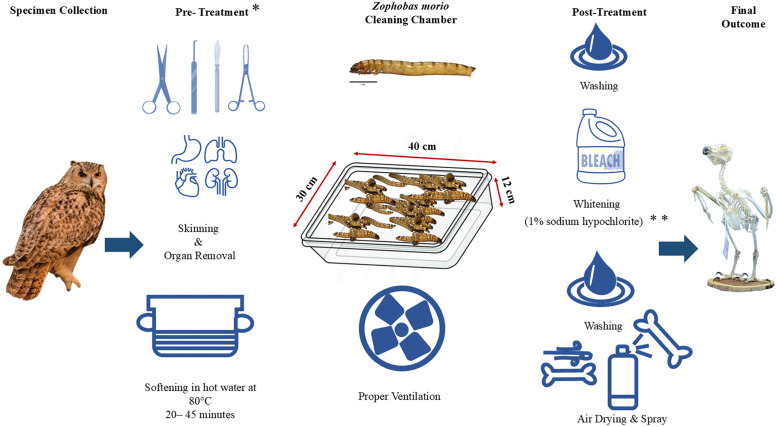
Experimental workflow for larval skeleton cleaning. Schematic overview of the main steps in the study, including specimen selection, pre-treatment, larval cleaning using superworms, and final skeletal preparation. Original photographs captured by the authors and created for this study. * The pre-treatment step depends on the size of the specimen. For small specimens, only skinning and organ removal were performed; however, for medium and large specimens, an additional soften tissue process in hot water at 80°C was carried out. ** We used a 1% sodium hypochlorite (bleach) solution in accordance with MZFUM protocol, but we recommend against this practice due to the potential for bone damage. If bleaching is desired, we suggest using a less aggressive alternative, such as natural extracts (lemon combined with baking soda), to minimize the risk of bone damage [22].

We processed specimens of various sizes and taxonomic groups under controlled conditions in order to assess the use of superworms for skeletal cleaning, with particular attention to cleaning efficiency, safety, and the preservation of delicate skeletal features (Fig 1). To provide context for evaluating the effectiveness and potential risks of superworm cleaning, we performed a parallel trial using a conventional boiling method. In this trial, a Marbled Polecat (*Vormela peregusna*) specimen was cleaned through boiling for 90 minutes followed by manual dissection.

In our study, superworms were sourced from a commercial insect supplier. Medium sized larvae (30–45 mm; approximately 40–50 day old and 0.25 grams) were selected. In our experiments, we used the containers (40 cm × 30 cm × 12 cm) that each received 300 grams of wheat bran as a supplemental substrate. The bran used was not fresh and served as a holding substrate, helping to reduce humidity around the larvae without providing a food source. During the experiment, the containers were kept at a controlled temperature (25–27°C) and relative humidity (40–60%) to optimize larval performance and minimize specimen degradation. The superworm colony was maintained in-house under controlled conditions. The colony consists of approximately 800–1000 adult specimens and was kept at the same condition. While superworms are primarily herbivorous, they can digest various types of food, including animal tissue [20]. However, our observations indicate that prolonged feeding on flesh may disrupt their molting process or increase mortality rates. To maintain larval health and allow their reuse, it is recommended to supplement their diet with plant-based materials, such as fruit or vegetable peels, after each cleaning session. In contrast, dermestid beetles can feed on the leftover animal tissue between experiments without exhibiting similar adverse effects [14]. The substrate is replaced weekly to ensure a clean environment. Under these conditions, we are able to harvest approximately 3.5 kilograms of larvae each month.

To evaluate the effectiveness of superworms, we selected eight species and categorized them by weight into three groups: Small (10–150 grams), medium (150 grams to 1.5 kilograms), and large (1.5 to 15 kilograms). These weights refer to the specimens after skinning and removal of soft tissues. All carcasses were donated to the Zoological Museum of Ferdowsi University of Mashhad (ZMFUM), sourced from the Department of Environment (DOE) or private contributors. Specimens were stored at −16°C for one to three days after arrival to reduce pathogen load. Based on personal experience, larvae were starved for 24 hours prior to use to stimulate feeding activity and increase their effectiveness in soft tissue removal.

### Pre-cleaning procedures, specimen preparation

To facilitate cleaning, all specimens were skinned and excess flesh and internal organs were removed. Larval density and preparation techniques were adjusted based on specimen weight. As the efficiency of skeleton cleaning varied depending on the larva to specimen ratio; however, we must be careful because there is always a trade-off between reducing cleaning time and avoiding damage to the specimens. To determine the optimal ratio, we used the same containers (40 cm × 30 cm × 12 cm) as a standard for all samples across three categories and filled each container with approximately 700 grams of larvae (based on our trials, this amounts of larvae represents the optimal larval density that can be accommodated in this container without causing overcrowding, which can negatively affect larval performance [20]. Prepared samples were placed in the containers along with the larvae. The weight of samples and larva to specimen ratio for each sample is presented in Table 1.

**Table 1:** Summary of cleaning time, specimen and larval weights, larva to specimen ratio.

Specimens	Specimen weight (gram)	Larval biomass (gram)	larva to specimen ratio cleaning	Time (hours)
Egyptian Rousette	9	700	77.78	1
House Mouse	30	700	23.34	2
Little Bittern	77	700	9.09	8
Alligator Gar	1030	2100	2.03	28
Eurasian Eagle Owl	723	2100	2.90	18
Rook	200	1400	7	12
Wild Cat ^*^	1910	4200	2.19	45
Gray Wolf ^*^	4200	5600	1.33	78

The table presents each processed specimen along with its post-preparation weight, the larval biomass, and the total time required for complete cleaning.

* Large specimens were divided into several containers.

**Small (House Mouse (*Mus musculus*), Egyptian Rousette (*Rousettus aegyptiacu*s), Little Bittern (*Botaurus minutus*))**:

Placed directly in larval containers after skinning and removing internal organs. The bat specimen used in this study was originally dried (mummified). To soften the tissue and facilitate larval cleaning, the mummified bat was immersed in hot water up to 80°C for 5 minutes before being placed in the larval container.

**Medium (Eurasian Eagle Owl (*Bubo bubo*), Rook (*Corvus frugilegus*), and Alligator Gar (*Atractosteus spatula*))**:

After skinning and removing internal organs, specimens were processed by softening tissues in hot water maintained as close as possible to 80°C for 20–30 minutes. This step was used to soften muscle tissue and aid in the removal of fats prior to larval exposure. Once the water reached 80°C, the specimens were placed in the water and the temperature was kept as close to 80°C as possible throughout the softening process. This duration was established through preliminary trials as the safe time range, minimizing the risk of bone damage. Following this pretreatment, medium specimens were placed in a single container. To maximize cleaning efficiency, each medium specimen was rotated every 6–8 hours into a new container with freshly starved larvae (starved for 24 hours), ensuring that each exposure was to actively feeding larvae.

**Large (Gray Wolf (*Canis lupus*) and Wild Cat (*Felis silvestris*))**: After skinning and removing internal organs, specimens were processed by softening tissues in hot water maintained as close as possible to 80°C for 30–45 minutes, then drained, air-dried for 5 minutes, and transferred to containers. Due to container size limits, large specimens were divided among several containers (Gray Wolf into four, Wild Cat into three). To adapt the rotation procedure used for medium specimens, while managing space and equipment constraints, we implemented a two-set system. Each portion of the sample was placed in Set A containers for 6–8 hours, then transferred to Set B (with freshly starved larvae) for the next 6–8 hours. During this time, the larvae in Set A entered a starvation phase to prepare for the next rotation. This two-set cycle was repeated until cleaning was complete. These steps were necessary due to space and container limitations; with larger containers or more available space, subdividing and rotating in this way would not have been necessary.

### Larval cleaning procedures

**Fig 2:**
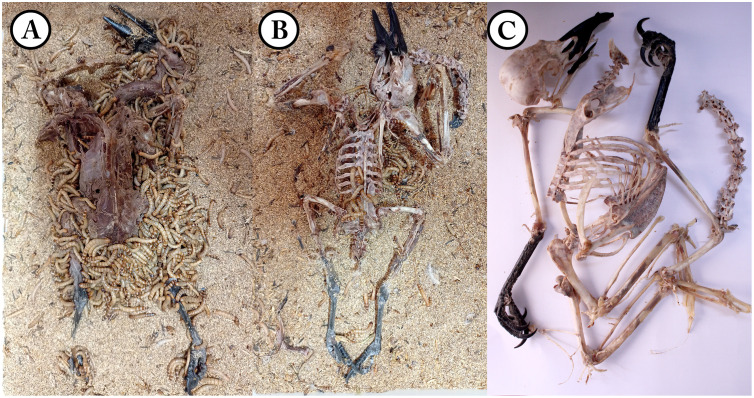
Sequential cleaning of a Hooded Crow (*Corvus cornix*) specimen by superworms (using larva to specimen ratio of 7). (A) Initial placement of the specimen into the larval container. (B) Specimen after 8 hours of larval cleaning, showing partial removal of soft tissues. (C) Specimen after 12 hours, with complete defleshing and intact skeletal elements preserved. Specimen was rotated every 6–8 hours into fresh containers. Original photographs captured by the authors and created for this study.

To enhance cleaning efficiency, the medium and large specimens were rotated every 6–8 hours into fresh containers, each containing newly starved, active larvae, to ensure continuous exposure to hungry individuals throughout the cleaning process (Fig 2). If a specimen became fully desiccated and the larvae could no longer consume hardened tissues (typically every 12–16 hours), the specimen was temporarily removed and tissues were softened in hot water for 5–10 minutes to rehydrate tissues, and then returned to the larvae for continued cleaning. Waste materials, such as removed soft tissues and larval frass, were regularly removed to maintain hygiene. Larvae exhibited higher activity in dark conditions; hence, light exposure was minimized to promote their activity [20].

After determining the optimal larva to specimen ratio from initial trials, we applied this ratio in follow-up tests on three additional small bird skulls. For these tests, the number of larvae was adjusted to achieve the targeted ratio in the same standardized container setup as described above (S1 Table). This was done to further assess bone preservation at the proposed optimal ratio.

### Post cleaning procedures

**Fig 3:**
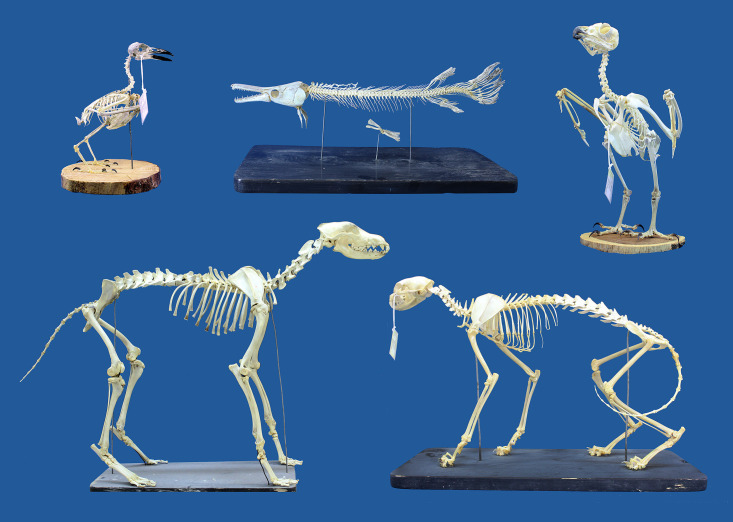
Cleaned skeletal specimens using superworms. Top from left to right: Rook, Alligator Gar, Eurasian Eagle Owl, Bottom from left to right: Gray Wolf, Wild Cat. Original photographs captured by the authors and created for this study.

After cleaning, skeletons were carefully removed from containers, and residual larvae or debris were manually cleared. Bones were rinsed with warm water to remove residual tissues. In accordance with MZFUM protocol, a brief immersion in a 1% bleach (sodium hypochlorite) solution was used; however, this step is not recommended, as bleach can potentially damage bone tissue. Skeletons were then thoroughly rinsed and air-dried. To protect and enhance appearance, the bones were coated with a clear gloss varnish spray (for CT-based analyses, bones should not be coated with varnish sprays). Each skeleton was labeled and added to the ZMFUM for research and education (Figs 3, S3–S7). Larval substrate was replaced after each cleaning session to maintain a clean environment, and larvae were fed fruit and vegetable residues between experiments.

## Result

As part of our investigation, we initially applied a traditional boiling and manual cleaning method to a Marbled Polecat (S1 Fig). This commonly used approach can cause deformation of delicate skeletal elements, such as rib bones, and often leads to dental loosening, making accurate tooth reseating difficult [23]. These issues underscore the limitations of conventional thermal and manual cleaning techniques.

### Optimal ratio and skeleton cleaning duration

Cleaning performance was assessed in relation to specimen size, larval density, and pretreatment conditions. The duration of skeletal cleaning was influenced by both the larval biomass and the size of the specimens. The Egyptian Rousette required the least time for complete cleaning, while the Gray Wolf took the longest (Table 1).

**Fig 4:**
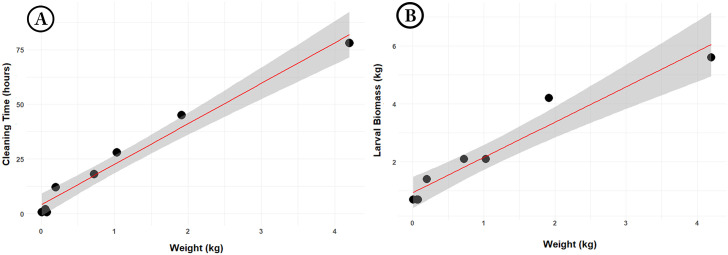
Correlation between specimen weight, cleaning time, and larval biomass by superworms. Positive correlations between post-preparation specimen weight and (A) cleaning time (r = 0.987, p < 0.001) and (B) larval biomass (r = 0.967, p < 0.001).

A strong positive correlation was observed between the specimen’s post-preparation weight and both cleaning time (Fig 4A, Table 1) and required larval biomass (Fig 4B, Table 1). For the small specimens, larval cleaning effectively removed all soft tissues; however, some fragile skeletal elements, particularly thin cranial and rib bones, suffered partial damage or consumption during the process for two samples of House Mouse and Egyptian Rousette (S2 Fig). No similar damage was observed in the medium and large specimens, nor in Little Bittern processed using a larval to specimen ratio of 9. Notably, even the delicate fish skeleton remained intact, with larvae effectively removing soft tissues within the skull without compromising bone structure.

There is an inherent trade-off between minimizing cleaning time and avoiding damage to delicate skeletal elements. Our results showed that very high larva to specimen ratios (such as 77.78 and 23.34 for House Mouse and Egyptian Rousette, respectively, Table 1) allowed for rapid cleaning but resulted in damage to fragile bones (Fig 4). In contrast, a lower ratio, such as ratio 9 for Little Bittern, prevented bone damage, but led to longer cleaning times (Fig 4). Through several trials, we suggested that a ratio between 10 and 15 achieves a balance, combining efficient cleaning with preservation of delicate skeletal structures. To confirm that using this ratio for small specimens does not cause damage to fragile bones, we applied the 10–15 ratio to three additional bird skulls in the small category, all of which were thoroughly cleaned without any observable bone damage (S1 Table).

## Discussion

The preparation of skeletal specimens is one of the fundamental aspects of zoological research and museum curation [1,2]. Careful cleaning preserves anatomical integrity and improves scanning accuracy for digital archiving and 3D modeling, which are increasingly used in modern museum science [24]. Thus, it is essential to adopt cleaning methods that are efficient, environmentally sustainable, while minimizing damage to the delicate skeletal structure.

Traditional methods, such as burial, enzymatic digestion, and chemical treatment, have been used but carry significant drawbacks [3]. These include the risk of irreversible damage to fragile bones, high operating costs, and environmental hazards associated with chemicals [3,9]. Biological cleaning using dermestid beetles is widely regarded as the preferred alternative due to its precision and minimal mechanical damage [14]. However, dermestid beetles present challenges in containment and management [16]. Since they complete their entire life cycle within the colony, posing risks of unnoticed egg laying or escape that threaten museum collections [25,26].

### Reduced environmental and museum risk

Cleaning with superworms larvae reduces the risk of museum infestation compared to dermestid beetles. Because only the larval stage is used for cleaning, adult emergence, reproduction, and egg-laying within skeletal samples are completely eliminated. Larvae require physical isolation to pupate, meaning that metamorphosis is effectively prevented under group-rearing condition [27]. This biological feature eliminates the risk of uncontrolled colony establishment within museum collections and simplifies colony management. Superworms also pose fewer risks of accidental escape. Adult beetles possess fully developed wings; however, flight is extremely uncommon and, when observed, is limited to short, weak movements [28,29]. Based on our observations during several years of superworm breeding, flight attempts occurred exclusively under acute stress, and these instances were brief and poorly sustained. Consequently, the strict containment measures required for dermestid beetles, such as sealed glass or metal enclosures, are unnecessary [14]. Furthermore, adult superworms feed primarily on moist or decaying organic matter and are not known to feed on keratinous materials such as skin or feathers, whereas adult dermestid beetles may attack preserved integumentary structures and damage valuable museum collections when they escape and hide in storage facilities [18,20,30].

### Biological and practical features of superworms

Superworms exhibit several biological characteristics that enhance their suitability for skeletal cleaning applications. The larval stage typically lasts 10–12 weeks, which is considerably longer than the 5–7 weeks reported for dermestid beetles (Dermestes spp.) [14,20]. The prolonged developmental period extends the duration of active larval feeding, which is more effective stage for tissue removal.

In addition, superworms reach body lengths of approximately 50–60 mm, substantially larger than dermestid larvae [20]. Their larger size is associated with stronger mandibles and a greater capacity for removing soft tissues, which facilitates the defleshing of specimens with substantial remaining musculature. These biological traits contribute to the effective performance of superworms in skeletal preparation contexts.

Beyond biological features, superworms also present several practical advantages for specimen preparation workflows. As they are widely used in the pet and feed industries, superworms are readily obtainable from commercial and domestic breeders [20,21], allowing for straightforward acquisition without extensive preparatory requirements. Moreover, they can be maintained under relatively simple environmental conditions, and colony establishment does not require highly specialized infrastructure [15,20].

### Optimizing cleaning efficiency

Our results emphasize the importance of the larva to specimen ratio in skeletal cleaning, revealing a trade-off between minimizing cleaning time and protecting fragile bones from damage. We suggest that a ratio of 10–15 larvae per gram offers an effective balance, combining efficient tissue removal with minimal risk of bone damage. Due to container size limitations in our experimental design, lower larva to specimen ratios were used for medium and large specimens, resulting in longer cleaning times (Table 1). In these cases, repositioning specimens and transferring them to fresh containers with newly starved, active superworms every 6–8 hours appeared to accelerate cleaning and partially compensate for the lower ratio. For optimal efficiency with medium and large specimens, we recommend using larger containers to allow the optimal larva to specimen ratio, which may further reduce cleaning times and the need for frequent repositioning. It is suggested that the cleaning process take place in well-ventilated areas with adequate airflow to maintain hygiene.

## Conclusion

Altogether, these findings demonstrate that superworms provide an adaptable, and effective alternative for skeletal preparation in museum and research settings. This method minimizes the risk of contamination and infestation for collections and aligns well with sustainable museum curation practices. By offering efficient cleaning, reliable preservation of specimen integrity, and straightforward, low-risk maintenance, the superworm method stands out as a practical, safe, and sustainable choice for skeletal cleaning in museum and scientific contexts.

## Supporting information

S1 Table Larva to specimen ratio optimization for bird skull cleaning. Larva to specimen ratio, skull weight, and cleaning duration for three bird skulls cleaned using superworms.
(DOCX)

S1 Fig Skeletal effects of the boiling method on a Marbled Polecat specimen. Deformation of rib elements caused by excessive heat during manual cleaning using boiling, resulting in warping of the thoracic structures. This illustrates a key risk associated with boiling defleshing techniques. Original photographs captured by the authors and created for this study.
(TIF)

S2 Fig Skeletal damage in small specimens caused by larval cleaning. Cranial damage observed in a bat (*Rousettus aegyptiacus*) specimen following larval cleaning. The zygomatic bone beneath the orbital cavity is fractured, and minor surface erosion is visible on the ventral side of the skull. Such damage highlights the limitations of using superworms for small and fragile specimens, where delicate skeletal elements are more vulnerable to feeding activity. Original photographs captured by the authors and created for this study.
(TIF)

S3 Fig Cleaned skeletal specimens using superworms. Alligator Gar. Original photographs captured by the authors and created for this study.
(TIF)

S4 Fig Cleaned skeletal specimens using superworms. Eurasian Eagle Owl. Original photographs captured by the authors and created for this study.
(TIF)

S5 Fig Cleaned skeletal specimens using superworms. Rook. Original photographs captured by the authors and created for this study.
(TIF)

S6 Fig Cleaned skeletal specimens using superworms. Wild Cat. Original photographs captured by the authors and created for this study.
(TIF)

S7 Fig Cleaned skeletal specimens using superworms. Gray Wolf. Original photographs captured by the authors and created for this study.
(TIF)
