# Supplementary material for: A practical and safe alternative method for skeletal cleaning for museum specimens using superworms (Zophobas morio)
Source: PLoS One. 2026 Jul 1;21(7):e0349669. doi: 10.1371/journal.pone.0349669 (PMC13322520; doi:10.1371/journal.pone.0349669)
Supplement: S1 Table — Larva to specimen ratio, skull weight, and cleaning duration for three bird skulls cleaned using superworms. (DOCX) [file pone.0349669.s001.docx]

| Specimens | Specimen Weight  (Post-preparation)  (gram) | Larva to specimen ratio | Cleaning Time  (hours) |
| --- | --- | --- | --- |
| Little Owl Skull | 11.88 | 14.93 | 4.5 |
| Little Owl Skull | 10.67 | 15.94 | 4.5 |
| Spotted Owlet Skull | 10.38 | 16.37 | 3.45 |
