# Supplementary figures and images for: A practical and safe alternative method for skeletal cleaning for museum specimens using superworms (Zophobas morio)
Source: PLoS One. 2026 Jul 1;21(7):e0349669. doi: 10.1371/journal.pone.0349669 (PMC13322520; doi:10.1371/journal.pone.0349669)

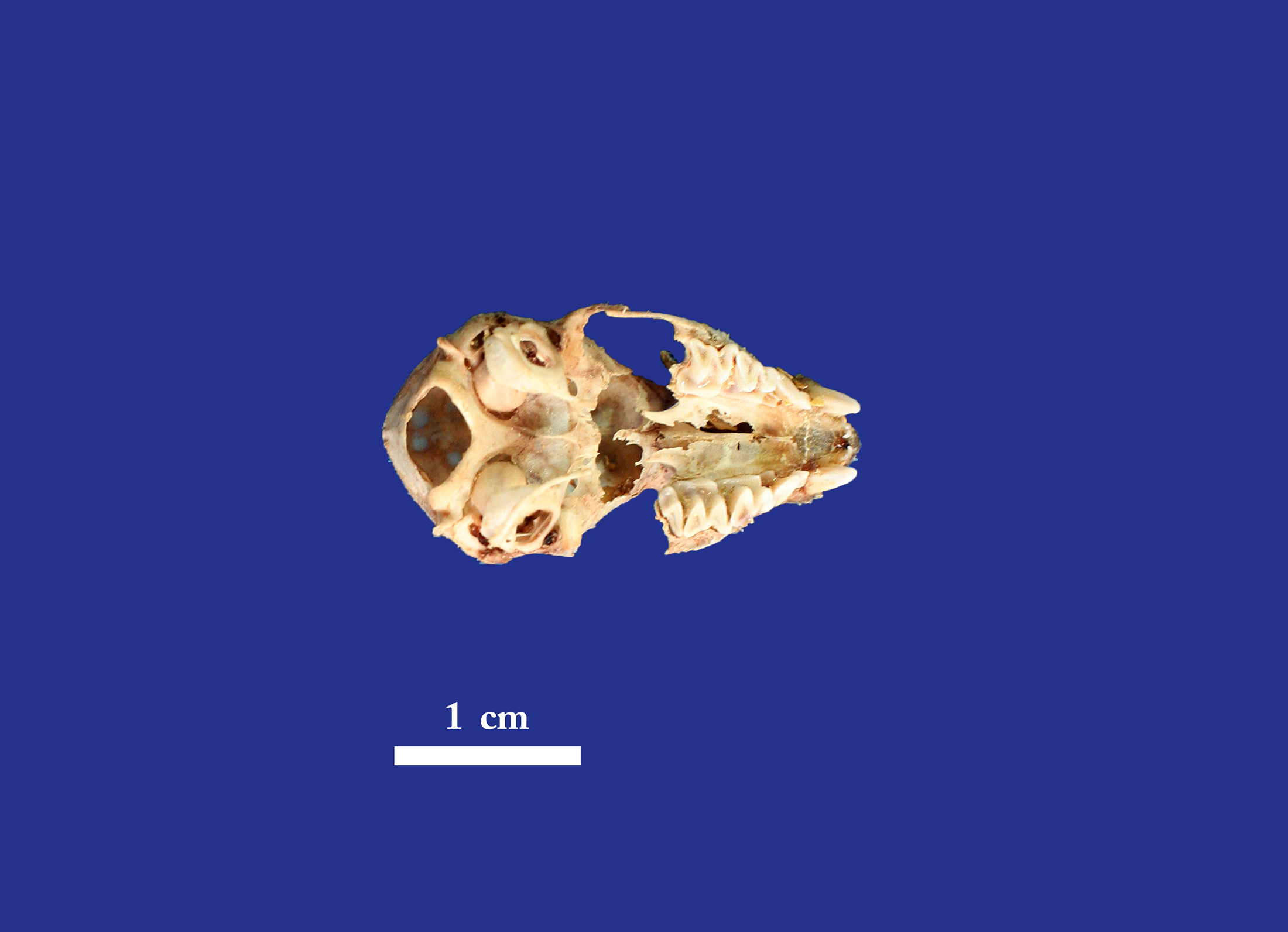

Supplement: S1 Fig — Deformation of rib elements caused by excessive heat during manual cleaning using boiling, resulting in warping of the thoracic structures. This illustrates a key risk associated with boiling defleshing techniques. Original photographs captured by the authors and created for this study. (TIF) [file pone.0349669.s002.tif]

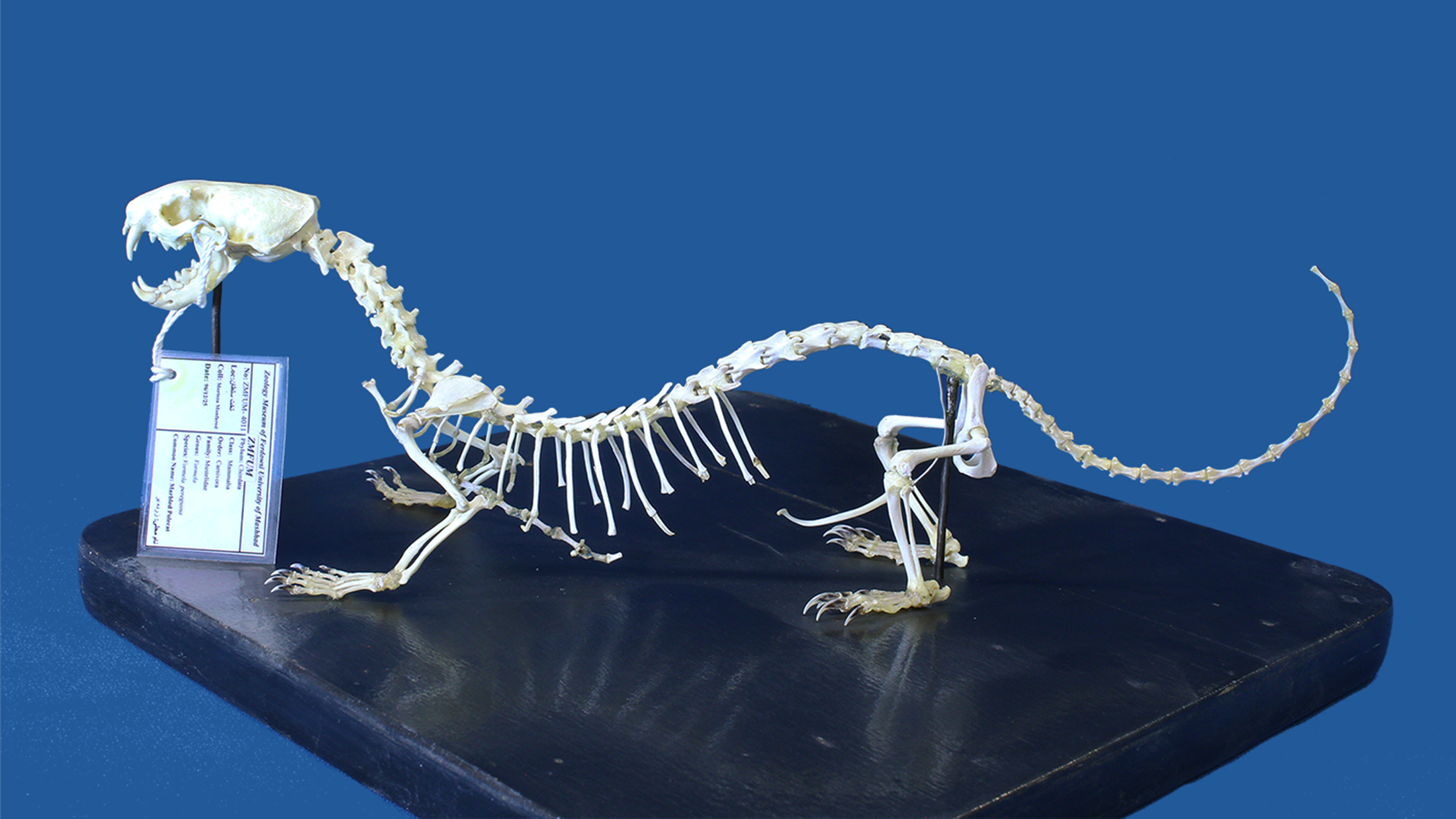

Supplement: S2 Fig — Cranial damage observed in a bat (Rousettus aegyptiacus) specimen following larval cleaning. The zygomatic bone beneath the orbital cavity is fractured, and minor surface erosion is visible on the ventral side of the skull. Such damage highlights the limitations of using superworms for small and fragile specimens, where delicate skeletal elements are more vulnerable to feeding activity. Original photographs captured by the authors and created for this study. (TIF) [file pone.0349669.s003.tif]

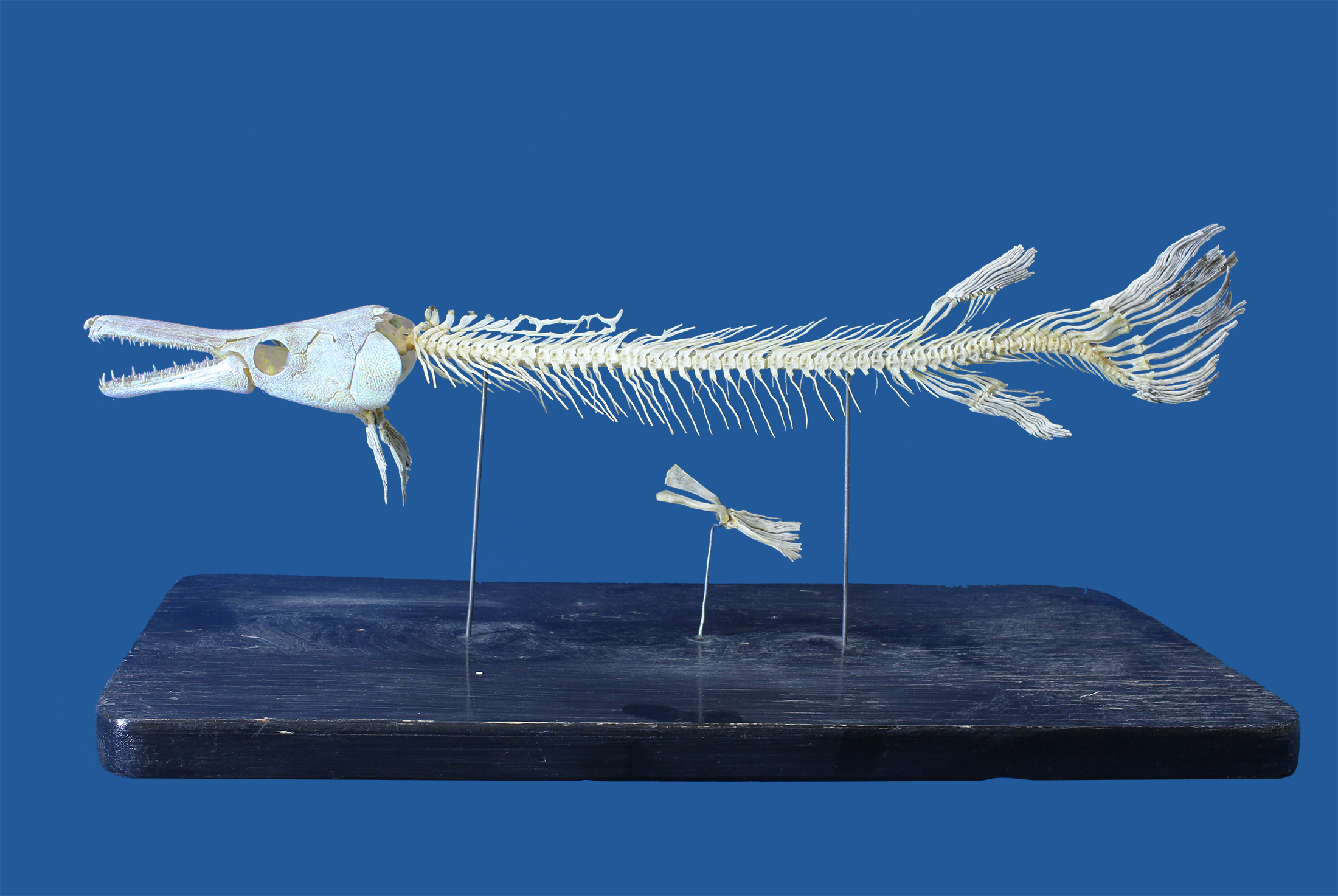

Supplement: S3 Fig — Alligator Gar. Original photographs captured by the authors and created for this study. (TIF) [file pone.0349669.s004.tif]

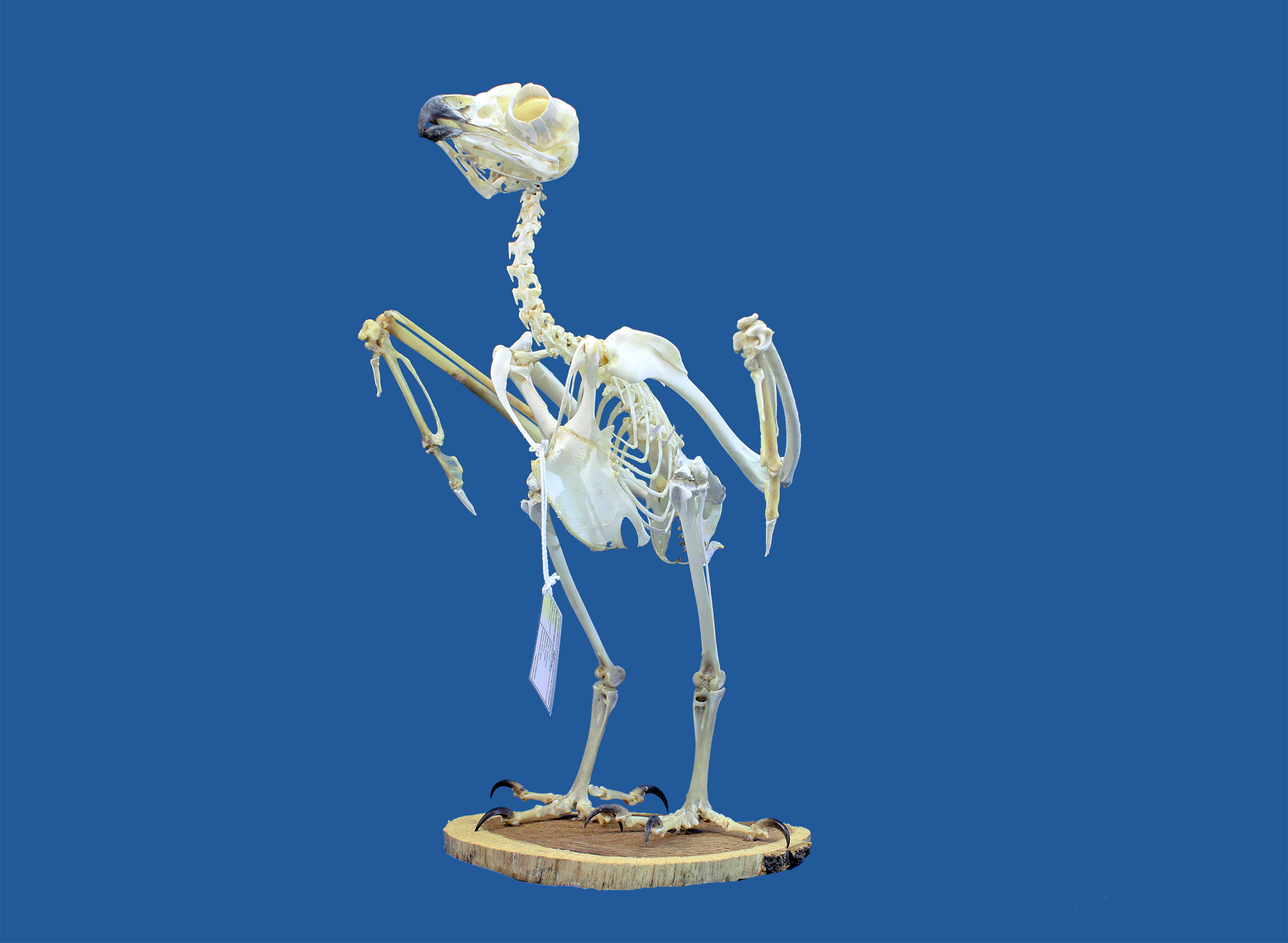

Supplement: S4 Fig — Eurasian Eagle Owl. Original photographs captured by the authors and created for this study. (TIF) [file pone.0349669.s005.tif]

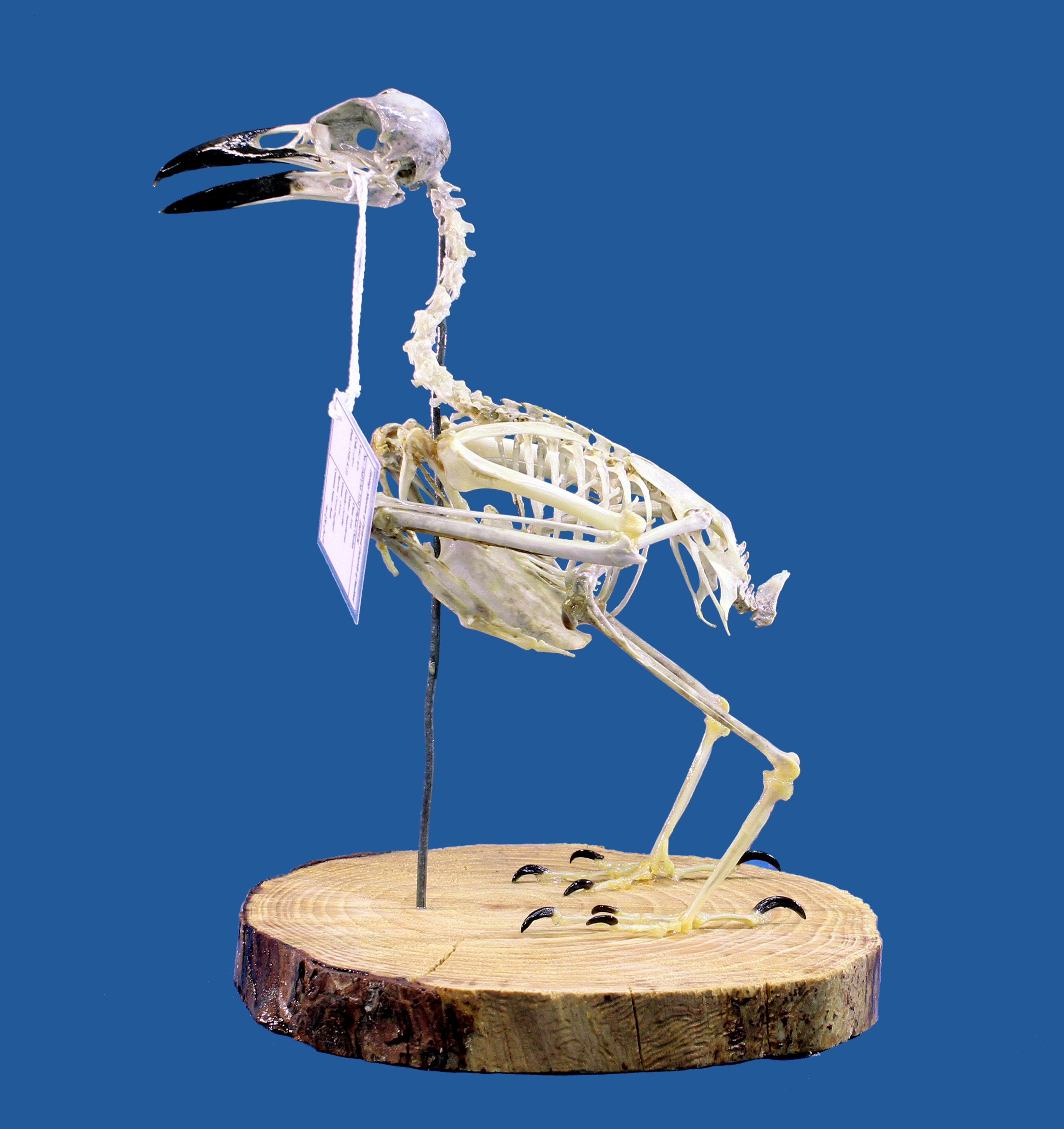

Supplement: S5 Fig — Rook. Original photographs captured by the authors and created for this study. (TIF) [file pone.0349669.s006.tif]

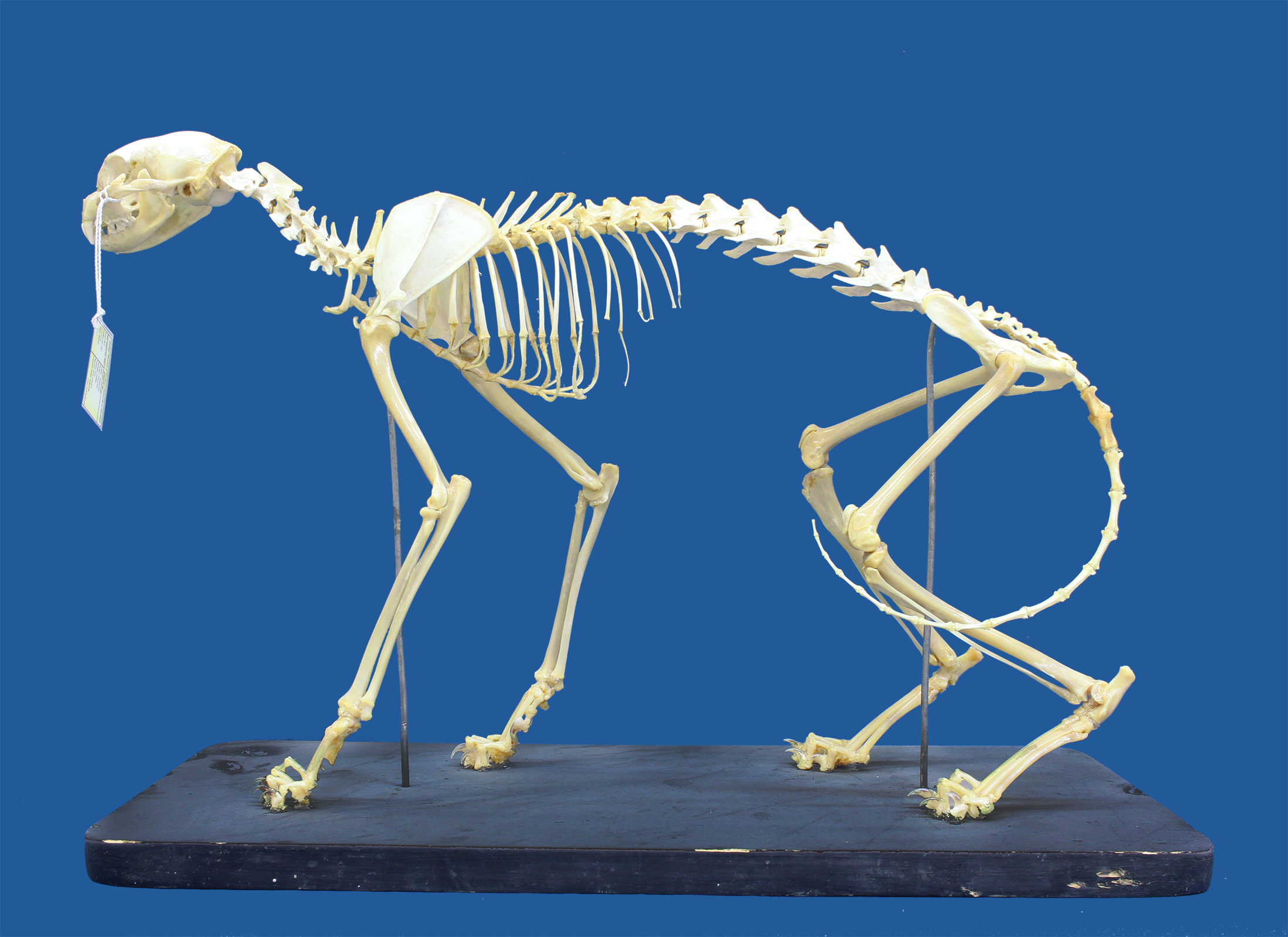

Supplement: S6 Fig — Wild Cat. Original photographs captured by the authors and created for this study. (TIF) [file pone.0349669.s007.tif]

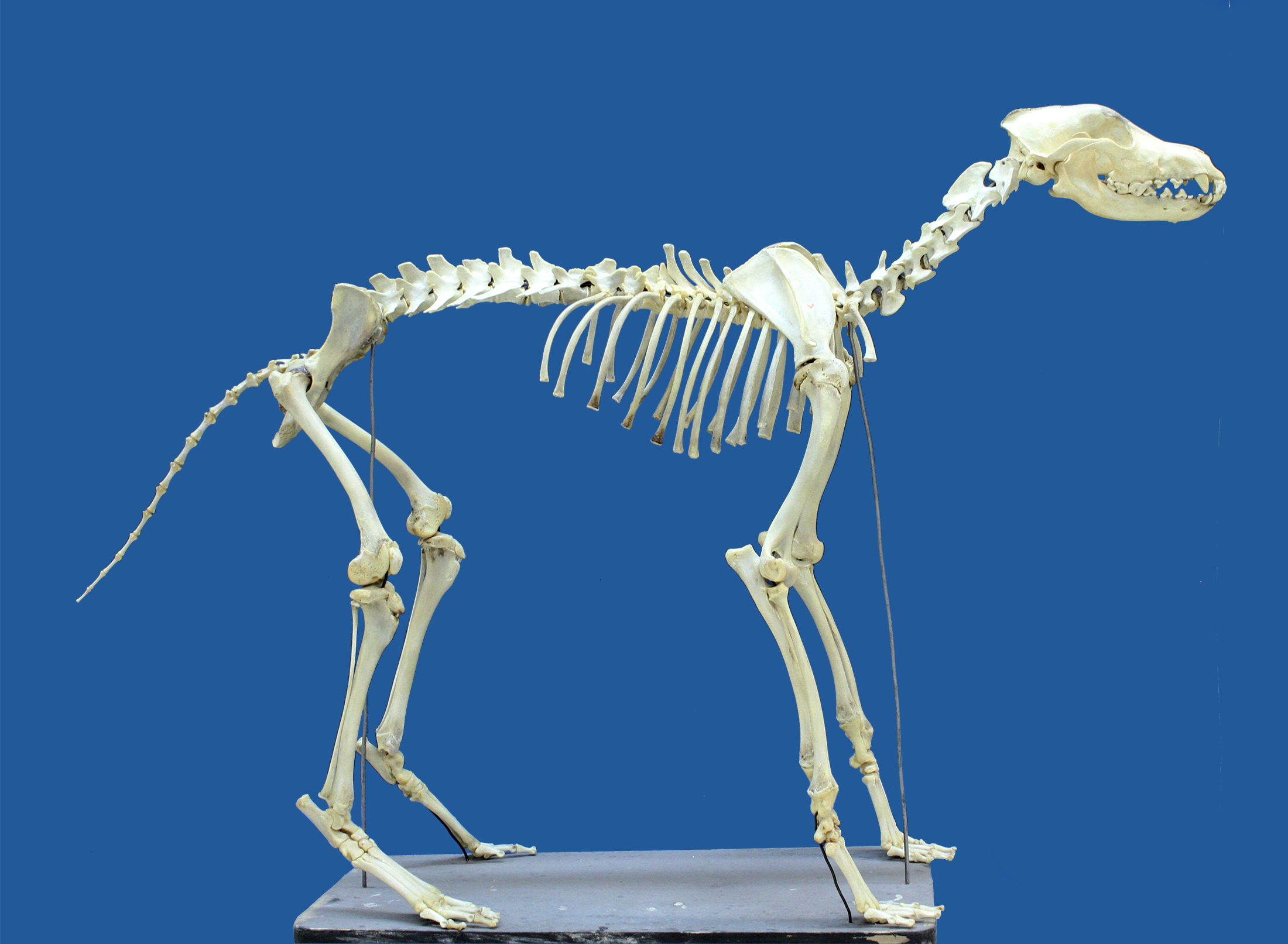

Supplement: S7 Fig — Gray Wolf. Original photographs captured by the authors and created for this study. (TIF) [file pone.0349669.s008.tif]
