# Supplementary material for: A practical and safe alternative method for skeletal cleaning for museum specimens using superworms (Zophobas morio)
Source: PLoS One. 2026 Jul 1;21(7):e0349669. doi: 10.1371/journal.pone.0349669 (PMC13322520; doi:10.1371/journal.pone.0349669)
Supplement: Attachment — Submitted filename: Response.docx [file pone.0349669.s009.docx]

Dear Editor, dear Reviewers,

We are pleased to resubmit a revised version of our manuscript, **“A practical and safe alternative method for skeletal cleaning of museum specimens using superworms (Zophobas morio)”.** We sincerely thank the reviewers for their positive assessment and constructive comments. We have addressed and responded to all comments, as detailed below.

Notably, we rewrote the section of the manuscript concerning the larva to specimen weight ratio, which we believe caused significant confusion in the original submission. By revising this section, we believe we have addressed the major concerns raised by the Editor and Reviewer 1. We hope that the Editor and Reviewers will find the revised manuscript suitable for publication in PLOS One.

With kind regards, on behalf of the authors,
**Morteza Monfared**

**RESPONSES TO AE AND REVIEWER COMMENTS**

**Editor Comments**

#1 - The ratios of larvae to specimen mass are inconsistent between the abstract, figures, tables, and text, and must be standardized throughout

***Authors’ Response****:* We agree that the original presentation of these data was confusing and lacked a consistent format throughout the manuscript. This issue was also pointed out by the first reviewer. In the revised version, we have thoroughly reworked this section to improve clarity and consistency. Specifically, we have revised the description of the larva-to-specimen ratio in the Methods (lines 146–150) and Results (lines 224–234) sections. We have also modified Table 1 to clarify this information and added new results obtained using the optimal ratio to the Supplementary Material as Table S1.

#2 - The starvation period for larvae should be supported by experimental evidence or properly referenced; otherwise, the claim that it improves cleaning efficiency should be removed or rephrased.

***Authors’ Response****:* Based on our personal experience, this short starvation period noticeably affects the larvae’s cleaning performance. However, since we do not have data to support this here, we have rephrased this part to indicate that we used this approach and propose it as a suggestion, please see the lines 131-132.

#3 - The boiling process also requires a clear rationale and description, as it poses potential risks to bone integrity. If maintained, the authors should explain why boiling was chosen, how its duration was determined, and why alternatives such as soaking were not preferred.

***Authors’ Response****:* We have already addressed this comment in the revised manuscript. To clarify, by “boiling” we did not mean the traditional boiling step. Instead, we soaked the samples in hot water at temperatures up to 80°C to gently soften the tissues and accelerate the cleaning process. To better reflect this procedure and avoid confusion, we have replaced the term “boiling” with “soaking” throughout the text.

#4 - The description of specimen weights should clarify whether values refer to intact or partially processed specimens, and repeated or redundant information should be reduced.

***Authors’ Response****:* These weights refer to the specimens after skinning and removal of soft tissues. We added to the text, please see line 127-128.

#5 - In the results and discussion, several statements need revision or better support. The claim that Z. morio is less damaging than dermestids beetle conflicts with observations of bone damage within twelve hours. Assertions about reduced costs, faster processing, or operational efficiency are not supported by data and should either be substantiated or removed.

***Authors’ Response****:* We rewrote most of the Discussion section based on the comments from the first reviewer. In the revised version, we now explain more clearly the advantages of using superworms in terms of speed and operational efficiency. We also removed the parts related to cost, as in our experimental setup we could not directly compare the costs of the two approaches.

#6 - Many passages require appropriate citations, and a few references are inaccurate or unrelated to the arguments made. The section introducing the marbled polecat and its cleaning method should appear earlier, preferably within the methodology.

***Authors’ Response****:* regarding the reference part, we worked on it and we added more appropriate references and regarding the part of the marbled polecat, thanks for pointing this out, we move this part into the method (lines 101-105) and result part (lines 200- 204).

#7 - The title should also reflect the actual scope of the work, emphasizing that the technique is particularly effective for medium and large specimens. The reviewers suggest a revised title such as “Efficient skeletal cleaning using superworms (Zophobas morio larvae): a practical alternative method for medium and large museum specimens.”

***Authors’ Response****:* We have changed the title based on the revised version of the manuscript and the identification of the optimal ratio, under which no damage to delicate bones was observed. Please see the description of the optimal ratio in the Methods (lines 146–150), Results (lines 224–234), and Discussion (lines 292–298) sections.

**Reviewer 1**

#1 - Line 57 vertebrates or vertebrate species

***Authors’ Response****:* It’s done.

#2 - Line 64 “a long time” add some more detail – decades, over 100 years

***Authors’ Response****:* It’s done.

#3 - Line 77 “exciting” should be existing

***Authors’ Response****:* It’s done.

#4 - Line 78 (and others) you already gave the Latin name, you do not need to keep providing it.

***Authors’ Response****:* It’s done.

#5 - The ratios that you use for larva-to-specimens is confusing, inconsistent, and not in the right format. These sections need to be rewritten. Figure 1 says 7:1 but the abstract says 77.78:1 and line 140 says 10 to 70. Line 151 is wrong and the ratios don’t match table 1.

***Authors’ Response****:* Thank you for pointing out the inconsistencies regarding the larva to specimen ratios. We agree that the original presentation of these data was confusing and did not maintain a consistent format throughout the manuscript. In this revised version, we have thoroughly reworked this section for clarity and consistency.

Specifically, we have revised the description of larva to specimen ratio in the Methods (lines 146–150) and Result (lines 224–234) section. We also modify the Table 1 to make this part more clear and we added the new result using the optimal ratio in the supplementary as table S1.

#6 - Line 112 were the super worms removed from the wheat bran to starve them?

***Authors’ Response:*** The wheat bran used as a substrate for the superworms was not fresh but had been recycled multiple times through previous rearing cycles. Consequently, its nutritional content was substantially reduced and it could not effectively serve as a food source for the larvae. We therefore used this bran primarily as a holding substrate, which also helped reduce humidity around the larvae. For the purposes of this study, the larvae were considered to be in a state of starvation for at least 24 hours before being placed in the cleaning containers, ensuring that they were highly active and motivated to consume the soft tissues of the specimens. This information has been added to the manuscript in lines 112–114. Moreover, we rephrased this section to present it as a recommendation for increasing the efficiency of superworms in skeletal cleaning.

#7 - Line 114 “this short starvation period (appeared to) stimulate the larvae”

***Authors’ Response****:* It’s added.

#8 - Line 115 what study or example are you using to claim significantly improved efficiency. Did you test with and without having a day of starvation? You do not know they were ravenous.

***Authors’ Response****:* Based on our observations during the experiments, we found that a brief starvation period of approximately 24 hours noticeably increased larval activity, thereby enhancing both the speed and efficiency of the cleaning process. This short starvation period ensured that all larvae were actively engaged in cleaning when introduced to the samples. Based on our personal experience, larvae were therefore starved for 24 hours prior to use to stimulate feeding activity and increase their effectiveness in soft tissue removal. This point has been included in the manuscript as a recommendation based on our observations (lines 131–132).

#9 - Line 117 and 102 are essentially identical, the same sentence stating we used eight different specimens shows up in lines 28, 91, 102, 117, 175. Cut out some of the repetition.

***Authors’ Response****:* The repitations were removed

#10 - Line 119 are the weights listed for intact specimens or are they the weights after skin and tissue removal? The groups can just be small, medium, large, you can cut “-sized specimens”.

***Authors’ Response****:* Yes, the weights reported in the manuscript refer to specimens after skin removal for all samples and after soft tissue removal for large samples. This clarification has been added to the manuscript in line 127.

#11 - Line 131 boiling a specimen, which could be considered a form of maceration, has the potential to damage bones. What it the justification for boiling these specimens, why are the risks not addressed, and how was the length of time determined? If boiling was used to soften tissue, why did you not just soak it in water like you did with the mummified bat, or conversely boil the bat? A properly skinned and cleaned Eurasian Eagle Owl should not have a significant amount of flesh remaining.

***Authors’ Response***: we totally agree with you and in the manuscript, by “boiling” we did not mean the boiling step used in the traditional method. Instead, the samples were soaked in hot water (at temperatures up to 80 °C) solely to soften the tissues and accelerate the cleaning process. To better reflect this procedure, we have replaced the term “boiling” with “soaking” in the manuscript.

#12 - Line 138 samples would imply that you only put part of the specimen in the container, is that true?

***Authors’ Response***: For large specimens only, we divided the samples into several smaller pieces so that they could fit into the containers, as the same type of container was used for all specimens.

#13 - Line 145 Wouldn’t the wheat bran serve as an additional source of food, which would run counter to the rotation to keep them hungry and active?

***Authors’ Response***: As we explained in the comments 6, we used the not fresh bran that could not effectively serve as a food source for the larvae.

#14 - Line 146 if the specimens were “fully submerged” why did they need to be repositioned and how could you check the process? The specimen in Figure 2 is not fully submerged

***Authors’ Response***: Yes, the small samples were fully submerged, whereas this was not the case for the medium and large samples due to the size of the containers. We have revised the text accordingly and now use the term “submerged” instead of “fully submerged.”

#15 - Line 175-177 this is not a result, degree delete

***Authors’ Response****:* It’s done.

#16 - Line 189 is not worded correctly, you added the larval biomas so “indicating that larger species require longer cleaning times and have greater larval biomass” doesn’t make sense. Possibly require larger biomas?

***Authors’ Response****:* Yes, it is the larger biomass. Thank you for pointing this out; this has been corrected in the manuscript.

#17 - Line 191 is redundant, just use small specimens you don’t need to list the species as they were already established

***Authors’ Response****:* It’s done.

#18 - Line 192 in your abstract and introduction (lines 23-27, 37, 65) you claim that superworms are better and safer but here you are saying they are damaging the bones and that damage is happening within 12 hours. That does not seem safer or better than dermestids. Line 72 “Dermestid beetles efficiently remove soft tissues while preserving the delicate bone structures”

***Authors’ Response****:* We have corrected this part in the text. Indeed, damage occurred in the small specimens when suboptimal conditions were used. By applying the optimal larva-to-specimen ratio, we overcame this issue, and no damage was observed in the small skulls tested when using a ratio between 10 and 15.

#19 - Line 198 where is your evidence that boiling “accelerated the process”

***Authors’ Response****:* Based on our experience during the experiments, we compared the cleaning process for large and medium-sized samples with and without soaking in hot water. For example, in the case of a hyena skull, we found that pre-soaking significantly accelerated the cleaning process. Specifically, soaking reduced the cleaning time by approximately 40%, indicating that this step can substantially improve efficiency.

#20 - Line 199 boiling for that duration wouldn’t cause visible damage but it can cause long term damage

***Authors’ Response****:* We fully agree with this point, and one of the advantages of our approach compared to the traditional boiling method is that it does not involve actual boiling. As explained in responses to Comments 6 and 7, we only soaked the samples in hot water, a step that did not cause any damage to the specimens.

#21 - Line 203 “providing an advantage over conventional methods that often require drilling or sawing” This needs a citation and as far as I know is not a standard practice for any of the other cleaning methods you listed.

***Authors’ Response****:* In some museums, for example the Museum of FUM, we have observed such cases. However, because we could not find suitable references to support this statement, we have removed this part from the manuscript.

#22 - Line 214 this is a comment, not a needed change but for improved scanning accuracy you should not be covering the specimen in a varnish.

***Authors’ Response****:* Thanks for your comments, it has been added.

#23 - Line 214-15 this needs a citation

***Authors’ Response****:* It’s added.

#24 - Line 218-219 the bones were damaged using superworms, operating costs were not addressed anywhere in the manuscript and your method also includes both manual dissection and maceration steps. This claim is not supported.

***Authors’ Response****:* This part is removed in the new revised version.

#25 - Line 221 why is this the first mention of the marbled Polecat and the alternate method used? This should have been addressed much earlier in the manuscript.

***Authors’ Response****:* Yes, you are correct. We have added this information to the beginning of the Methods section.

#26 - Line 224 missing citation.

***Authors’ Response****:* It’s added.

#27 - Line 228 the challenges are not “substantial” as evidenced by all the institutions using that method on line 70. The citation used explains how to safely contain dermestid beetles.

***Authors’ Response****:* Thanks for pointing this out, it is corrected in the new version.

#28 - Line 231 the citation used does not say this, in fact, it essentially says the exact opposite. This is also not true unless you are speaking about suppliers local to you, in which case that needs to be clarified.

***Authors’ Response****:* This error occurred during the referencing process, and we apologize for it. In this updated version, we have carefully reviewed the references and corrected the mistake.

#29 - Line 242 but dermestids can feed as adults, meaning their feeding period does not end.

***Authors’ Response***: That is correct; however, feeding activity during the larval stage is more effective compared to other stages. In our study, we used only the larval stage of the superworms, resulting in higher feeding efficiency compared to beetles, where both larvae and adults are involved. An additional advantage is that managing superworms, all at the same larval stage, is much easier and reduces the risk of contamination to museum collections.

#30 - Line 250 cost is not listed anywhere in this manuscript and how are you assessing these costs? Letting a specimen sit in dermestid beetles for slightly longer shouldn’t add additional costs.

***Authors’ Response***: We agree that it is difficult to directly assess the cost of the experiment. By “cost-effective,” we intended to convey that this approach is faster, as all the specimens are in the larval stage, making colony management easier and safer. It does not require post-processing steps after cleaning, nor does it involve removing eggs, which is particularly important for the safety of museum samples. Overall, this method is less time-consuming and requires less human intervention. However, as you correctly noted, we cannot directly assess the cost, and therefore we have removed these statements from the manuscript.

#31 - Line 256-258 you can feed dermestid beetles (or superworms) tissue removed from specimens, reducing the cost of food to 0.

***Authors’ Response***: As we explained in Comment 30, we have removed this part, along with other sections related to cost.

#32 - Line 252 there are no actual costs listed so how does the reader know what the costs are? Colony maintenance is not addressed in the manuscript, so how is the colony being maintained, are you buying new superworms every 10-12 weeks?

***Authors’ Response***: Regarding the cost-related comments, since we could not directly assess the actual costs at this time, we have removed all sections referring to cost-effectiveness from the manuscript to avoid any confusion. As for colony maintenance, we maintain the superworm colony in-house rather than purchasing new individuals every 10–12 weeks. To clarify this, we have added a detailed description of the colony maintenance protocol in the Methods section (lines 120–124).

#33 – Lines 262, 264 missing citations

***Authors’ Response***: This section is based on our direct observations from the experiments. We have rewritten it in the manuscript to make it clear that it is a suggestion derived from our experimental experience.

#34 - Line 266 this seems like it should have been mentioned much earlier in the manuscript and like an issue for an institution using this method. Dermestids do not require diet supplementation.

***Authors’ Response***: We have moved this section to the part that we talked about the colony maintenance (lines 120 -124).

#35 - Line 276 cost was not proven

***Authors’ Response***: The section discussing cost-effectiveness has been removed from this revised version of the manuscript, as the costs were not directly assessed or validated.

#36 - Line 280 this is the first mention of mealworms in the entire manuscript.

***Authors’ Response***: This part is removed in the new version.

#37 - Line 385 this only illustrates a risk for boiling, not for “traditional defleshing techniques” Are new worms being purchased regularly? Monitoring every 6-8 hours and repositioning seems like more work than using other methods (line 148) tissue getting to dry after 12-16 hours is not a problem for dermestids (155) line 39 contamination risk is not addressed

***Authors’ Response:*** We address your comments as follows:

**Line 385:** You are correct; the risk described applies to boiling, not to traditional defleshing techniques. We have corrected the manuscript accordingly, replacing “traditional defleshing techniques” with “boiling.”

**Colony maintenance:** We maintain the superworm colony in-house. Please refer to the Methods section, lines 120–124.

**Lines 148 and 155:** These points are not limitations of the method itself. For medium to large specimens, it is more convenient to use a larger container so that all parts of the specimen are fully accessible to the superworms. As shown in Table 1, the larva-to-specimen ratio for large specimens remains low but could be increased to the optimal ratio of 10–15. In our experiment, we deliberately used the same container size and the same number of larvae per container across all treatments to maintain a consistent ratio. Using a larger container allows for more superworms per sample, which accelerates the cleaning process and eliminates the need for repositioning or monitoring the samples every 6–8 hours, as the superworms have full access to all surfaces. The necessity of repositioning depends on the container size; in this experiment, using the same container size across all samples required repositioning to prevent tissue drying and ensure effective cleaning.

**Contamination risk (line 39):** This point has been added in the revised version of the manuscript (line 40).

**Reviewer 2**

This manuscript clearly shows a viable option for cleaning bone material in scientific collections, in which the bone characteristics of the specimens must be kept as complete as possible.
In this regard, the method using the larvae of the beetle Tenebrionidae Zophobas morio meets the desired characteristics for collections: it is efficient, takes as little time as possible, and causes the least damage to bone structures; it also reduces contaminating waste and has a low potential to become a pest in the collection.

#1 - However, as the authors point out, the method is particularly efficient for medium to large specimens, so perhaps this detail could be reflected in the title “Efficient skeletal cleaning using superworms (Zophobas morio larvae): a practical alternative method for medium and large museum specimens.”

***Authors’ Response:*** We are very pleased that you appreciated our work. Regarding the title of the manuscript, we have revised it to better reflect the content and findings of the updated version of the study.

#2 - In the same vein, it would be useful to compare the effectiveness of the larvae of the other species mentioned for smaller specimens against the larvae of the early stages of Z. morio to try to avoid the establishment of colonies of various species of darkling beetles.

***Authors’ Response:*** In this revised version, we recommend using the optimal larva-to-specimen ratio (10–15) for small samples. We have already tested this ratio on three small specimens and confirmed that using the optimal ratio caused no damage.

#3 - On the other hand, it is suggested that the authors seek less aggressive alternatives to sodium hypochlorite for bleaching specimens, as it has been documented that if it is not completely neutralized, it continues to act on the mineral matrix of the bone.

***Authors’ Response:*** In this revised version, we have included alternative approaches suggested in recent studies as less aggressive options for bleaching specimens, which could be considered instead of sodium hypochlorite (lines 305-307).
