# Supplementary material for: A practical and safe alternative method for skeletal cleaning for museum specimens using superworms (Zophobas morio)
Source: PLoS One. 2026 Jul 1;21(7):e0349669. doi: 10.1371/journal.pone.0349669 (PMC13322520; doi:10.1371/journal.pone.0349669)
Supplement: Attachment — Submitted filename: Response-to-Reviewers.docx [file pone.0349669.s010.docx]

Dear Editor, dear Reviewers,

We are pleased to submit the revised version of our manuscript, “A practical and safe alternative method for skeletal cleaning for museum specimens using superworms (*Zophobas morio*).” We sincerely thank the Editor and Reviewers for their thoughtful and constructive feedback. We have carefully addressed every comment and point raised, as detailed below. Notably, we have revised our terminology, an issue which was central to concerns with the original submission, and provided additional experimental details to clarify our methodology and improve reproducibility. We believe these major revisions address and resolve the key points raised, including those highlighted by academic editor and reviewer 1. We hope that the Editor and Reviewers find our revised manuscript suitable for publication in PLOS ONE.

With kind regards on behalf of the authors,

Morteza Monfared

**RESPONSES TO AE AND REVIEWER COMMENTS**

*The line numbers referenced in our responses correspond to the clean version of the manuscript without tracked changes.*

**Academic editors**

Editors’ comments to the author:

Thank you for submitting your manuscript to PLOS ONE. After careful consideration, we feel that it has merit but does not fully meet PLOS ONE’s publication criteria as it currently stands. Therefore, we invite you to submit a revised version of the manuscript that addresses the points raised during the review process. Based on Reviewer 1’s comments, the manuscript requires substantial revision before it can be reconsidered for publication. The review identifies significant shortcomings in methodological rigor, clarity, and evidentiary support, which currently undermine several of the manuscript’s main conclusions.

A central concern is the use of misleading terminology. Referring to water at 80 °C as “soaking” is inappropriate and must be corrected. The procedure should be described precisely, including how temperature was measured, whether it was maintained or allowed to cool, and the exact duration of exposure.

Several key conclusions are not supported by the experimental design. Claims that soaking accelerated cleaning or that repositioning specimens improved efficiency lack proper controls and therefore must be removed or clearly rephrased as descriptive observations rather than causal statements.

The comparison with dermestid beetles is also problematic. The manuscript does not provide comparable data to support claims of superior cleaning speed, and Table 2 includes inconsistent specimens and questionable citations. This comparison should be removed, substantially revised, or strongly qualified.

Methodological details are insufficient in multiple areas, including larval replacement, specimen repositioning, number of containers used for large specimens, monitoring intervals, and criteria for determining completion. These variables must be clearly defined to ensure reproducibility.

In addition, several numerical claims, particularly regarding the optimal use of 700 g of larvae and monthly larval production, are not adequately supported and appear inconsistent. These statements require justification or removal.

The manuscript also contains redundancy, unclear statements, inconsistent terminology (e.g., “samples” vs. “specimens”), and errors in scientific naming and citation. Tables and figures need revision to provide meaningful quantitative information and visual evidence that supports the claims made.c

In summary, major revision is required. The authors must address methodological gaps, remove or qualify unsupported claims, improve clarity and consistency, and correct all issues related to data presentation and citations before the manuscript can be reconsidered for publication.

***Authors’ Response****:* *We sincerely thank the Academic Editor for the careful consideration and feedback on our manuscript. We have thoroughly revised the manuscript to address all major concerns and bring the work in line with PLOS ONE’s publication criteria.*

***Misleading terminology***

*We acknowledge that referring to the pre-cleaning step as “soaking” at 80 °C was imprecise and potentially misleading. The reason we changed the term from "boiling" to "soaking" was that our process did not involve a true boil; however, we appreciate that "soaking" does not accurately reflect the conditions either. In the revised manuscript, we have completely rephrased this process to describe it as “softening tissues in hot water at 80 °C.” We now provide a precise methodological description, explicitly noting how temperature was measured and maintained, and the exact duration of exposure for each specimen type (please see methods, line 164-169 and 175-176).*

***Unsupported claims and experimental control***

*We have carefully reviewed all conclusions and have removed or rephrased any claims that were not directly supported by our experimental design or the available controls. For example, regarding the pre-treatment step and its impact on cleaning efficiency, we now provide a detailed explanation in the text of how the experiment was set up and specifically why this step is suggested to accelerate the process. For the tissue-softening step, we used a laboratory heater with a thermometer to maintain the water temperature at 80 °C for 20–30 minutes, with duration determined by preliminary trials to ensure effective softening and no bone damage. These specifics have been clarified in the text (please see method, line164-169). To evaluate the effect of this pre-treatment, we conducted a comparative trial with two wolf skulls of similar size: one underwent softening at 80 °C before larval exposure, the other did not. The pre-treated skull had about a 50% reduction in cleaning time and showed no bone damage (please see also our detailed response to Reviewer 1, comment 19").*

***Comparison with dermestid beetles***

*We totally agree that we cannot directly compare our results with those from the studies listed in Table 2 due to inconsistencies in specimen type and methodological details. The specimens referenced, which were cleaned by dermestid beetles, are drawn from various studies and do not directly match the species, specimen preparation, or cleaning protocols used in our work. As you and reviewer 1 noted, important methodological details such as dermestid pre-conditioning (e.g., starving, regular replacement) are not stated, making the cleaning durations difficult to compare. Therefore, all comparative claims of superior cleaning speed by superworms have been removed. We now in this revised version of manuscript only discussed the result that we got from our experiment.*

***Methodological clarity and numerical claims***

*We have expanded the Methods section to clearly define all important variables, we explained our setup and the limitation and the process that we did the experiment with details. We try in this version clearly define all procedures and variables, including larval replacement, specimen handling, rotation, monitoring intervals, use of containers, and completion criteria* *(please see the first part of method section and also in the response to the reviewer 1 comments 20 and 23, 24).*

***Clarity and consistency***

*The manuscript has been thoroughly edited to remove redundancy, clarify ambiguous statements, standardize terminology, and correct all errors in scientific names and citations. Tables and figures have been revised and now provide quantitative information and clear visual documentation directly supporting our claims.*

**Reviewer 1**

#1 - I would not consider 80 degrees C as soaking, 20-25 C is room temperature 100 c is boiling 80 is much closer to boiling than warm water. Changing the word boiling to soaking is deceptive.

***Authors’ Response****: Thank you for your comment and for highlighting issues with our terminology. We apologize for any confusion caused by our initial description.* *The reason we changed the term from "boiling" to "soaking" was that our process did not involve a true boil; however, we appreciate that "soaking" does not accurately reflect the conditions either. To clarify, our methodology involved softening the tissue by placing the specimens in water maintained at 80°C, using a laboratory heater and thermometer. Once the water reached 80°C, the specimens were placed in the water, and we kept the temperature as close to 80°C as possible throughout the soften process. This process was selected to soften muscle and connective tissues and to help remove fats, thereby improving the efficiency of subsequent larval cleaning. Based on your remarks, we acknowledge that "soaking" can be misleading and that "boiling" is not accurate for this process. In the revised manuscript, we now called this step as "softening tissue process in hot water* *at 80°C". We believe this terminology accurately reflects our procedure and provides clarity for readers.*

#2 - Line 28 It might just be the version I am seeing, but italicize the Latin name, remove larvae from the parentheses.

***Authors’ Response****: It’s done.*

#3 - Line 34 typo on grams

***Authors’ Response****: It’s done.*

#4 - Line 68 the citation added does not back up this claim, this is another improper citation

***Authors’ Response****: The better citation is added.*

#5 - Line 82 the Latin name needs to stay here, this is the first mention of superworms (after the abstract) but take larvae out of the parenthesis

***Authors’ Response****: It’s done.*

#6 - Line 87 “superworms do not pupate under crowded conditions” citation?

***Authors’ Response****: It’s done.*

#7 - Line 95 its not really samples, it's maybe species or sizes of species. In its current updated form, this sentence doesn’t really tell me anything

***Authors’ Response****: You are right, and in this new revised version this sentence is removed from the manuscript.*

#8 - Line 106 says the same thing as lines 81-83. The effectiveness of superworms was evaluated using a diverse range….

***Authors’ Respons****e: We have removed the redundant statement from the beginning of the Methods section.*

#9 - Line 110-113 this is a confusing sentence and it doesn’t say anything but a test was conducted, what were the results? It also doesn’t really fit into this section.

***Authors’ Response****: Our intention in this section was to introduce a comparative trial between a traditional non-biological method (boiling in hot water) and the superworm cleaning approach, in order to highlight the differences in risk and processing time. We have reworded this part of the Methods section to state the comparative aim more explicitly, and we have clearly indicated that the corresponding results—regarding risk of specimen damage—are presented in the Results section (see Line 99-102 in method and line 222-226 in Result). If you feel this information disrupts the methodological flow, we are also open to moving this description to the Results or Discussion section for improved clarity and readability.*

#10 - Line 118 replace samples with specimens or animals

***Authors’ Response****: It’s done.*

#11 - Line 123-125 this sentence is confusing and what does the (20) mean?

***Authors’ Response****: This sentence has been rewritten in the revised manuscript for greater clarity.*

#12 - Line 132 citation

***Authors’ Response****: It’s done.*

#13 - Line 133 I like how you address the longer term health of the colony and provide a solution but

#14 - line 32-33 make it sound like this may not be a great sustainable alternative to dermestid beetles. On the other had it may be a benefit because you could just feed them food scraps between specimens.

***Authors’ Response****: Thank you for your comment. We have added this point, with the reference, to the manuscript (see Lines 123-129). Additionally, we have revised all sections where we compare the maintenance of dermestid beetles with superworms to ensure clarity and accuracy throughout the text.*

#15 - Line 137 3.5 kilograms of larva each month seems like the wrong number, 1000 adult superworms would weigh 450g - 1.36kg, how are they producing 3.5kg of larva each month?

***Authors’ Response:*** *The weight of adult superworms does not directly correspond to the total larval biomass produced, as adults can reproduce multiple times each month under optimal conditions. Each female is capable of laying dozens of eggs per month, and with proper husbandry, a significant percentage of these eggs develop into larvae. We have been successfully maintaining and operating our superworm colony for several years, consistently producing up to 3.5 kilograms of larvae each month under suitable conditions.*

#16 - Line 142 check this sentence. I think a word is missing but hard to tell with the tracked changes on.

***Authors’ Response****: It’s corrected.*

#17 - Lines 159, 163, 166 these are the first mentions of these species, add the Latin names, also take out e.g, that is not an example those are the exact and only species you used.

***Authors’ Response****: It’s added.*

#18 - Lines 160 and 161 are contradictory, also was the mummified bat skinned before it was mummified, after, or not at all and did it happen before or after soaking it?

***Authors’ Response****:* *The bat specimen was already dried and mummified prior to any preparation in our study, and it was not skinned either before or after mummification. To soften the tissues and facilitate larval cleaning, we placed the intact mummified specimen in hot water at up to 80°C for 5 minutes before placing it in the larval container. This procedure has been clarified in the revised manuscript (see lines 158-161).*

#19 - Line 165 did you have a thermometer in the water to confirm? Was the water kept at the same temperature for 20-30 minutes or was it heated and then allowed to cool. Fresh tissue is already soft so I still don’t understand why this step was necessary, especially with the potential damage it could cause to the bones. How did you choose 20-30 minutes? Did you see a clear change in the tissue after that process? Was it falling off the bones?

***Authors’ Response****: Yes, we used a laboratory heater with a thermometer throughout the process to ensure that the water temperature was maintained as close to 80°C as possible for the entire 20–30 minutes period. The soften duration was determined through preliminary trials, during which we tested various time intervals and evaluated each sample for effective tissue softening and potential bone damage. We consistently found that the 20–30 minutes range was both safe and effective across different specimens, with the precise duration adjusted according to the size and maturity of each sample. We make it clearer in the text also (see lines 164-169). To assess the necessity and impact of this step, we conducted a comparative trial using two wolf skulls of similar size and weight: for one skull we used the soften process at 80°C prior to larval exposure, while the other was not. The skull subjected to the pretreatment process exhibited approximately a 50% reduction in cleaning time, with no observable bone damage or excessive tissue detachment. Based on these results, we included this pre-treatment step to significantly enhance the overall efficiency of the cleaning process without compromising specimen integrity.*

#20 - Line 168 how many containers were used for the large specimens? What is the value of using large specimens if you separate them out into multiple containers that might be comparable to the medium specimens

### ***Authors’ Response:*** *Due to the use of fixed-size containers (each measuring 40 cm × 30 cm × 12 cm) in our experimental design, large specimens could not be accommodated in a single container. As a result, each large specimen was divided into multiple containers, with each container receiving 700 grams of larvae. This amount of larvae represents the maximum larval density that allows for optimal humidity, temperature, and larval activity within one container (just based on the size of containers that we use in our experiments). For clarity, we have added this explanation to the manuscript (see lines 177-185 and in discussion in part “Optimizing cleaning efficiency”). For example, the Gray Wolf was divided among four containers and the Wild Cat among three containers. In our trials, attempting to fit more larvae into a single container led to overcrowding activity (please see reference 20), which reduced cleaning efficiency and created suboptimal conditions for larval health. Dividing large specimens in this way ensured consistent and effective cleaning across all samples.*

#21 - Line 169 based on the larva? Fix the tense used

***Authors’ Response****: It’s corrected.*

#22 - Line 171, 174 specimen not sample

***Authors’ Response****: It’s done.*

#23 -For table 1 to be useful you also need to capture the clean weight of the specimen to determine how much tissue was eaten/removed, which would tell you what the consumption rate was, and you could use that formula for any sized specimen. What is the explanation for why the alligator gar took 10 more hours than the Eurasian eagle hour when they had similar ratios? Make sure you capitalization is consistent. It also needs to be clarified how the times for the large animals in multiple containers worked. Were the containers all started at the same time for an animal or did you wait for one container to finish before starting the next one?

***Authors’ Response****: We answer each of your points in order:*

***Specimen Clean Weight and Consumption Rate:***

*We agree that recording the clean weight of each specimen would have allowed us to calculate the consumption rate and compare cleaning efficiency across different sizes. Unfortunately, in this study, we only measured the weight of the specimen after skinning, before cleaning began, and did not record the final clean (skeleton-only) weight.*

***Alligator Gar vs. Eurasian Eagle Owl Cleaning Times:***

*Although both the Eurasian eagle owl and alligator gar were medium-sized specimens, the eagle owl was about 300 grams lighter and had a slightly higher larva-to-specimen ratio (approximately 3 for the eagle owl versus 2 for the alligator gar), which may partially explain the faster cleaning time. Additionally, biological differences in tissue composition—such as tissue density and structure between birds and fish—may also contribute to the observed differences in cleaning duration.*

***Timing and Rotation in Large Specimen Cleaning:***

*For large specimens, the container size used in our experimental setup did not allow us to place an entire specimen in a single container. As a result, each large specimen was divided among several containers (e.g., the wolf sample was divided into four containers). For medium specimens, we used a rotation system in which the specimen was moved every 6–8 hours into a new container with freshly starved larvae (starved for 24 hours), ensuring each rotation used actively feeding larvae. However, due to space limitations in our lab, we could not rotate every portion of large specimens into new containers every 6–8 hours (as we have several containers for one sample, for example 4 containers for Wolf). Instead, we prepared two sets of containers (Set A and Set B, each with four containers for the wolf and three containers for Cat). All portions of the large specimen were placed in Set A simultaneously. After 6–8 hours, the samples were transferred to Set B (with freshly starved larvae). While Set B was active, the larvae in Set A entered a starvation period for the next 6-8 hours, to be prepared for the next rotation. This two-set rotation process continued until cleaning was complete. We used this approach due to constraints in space and available containers, which affected both the achievable larva-to-specimen ratio (resulting in a lower ratio for large samples) and overall cleaning time (leading to longer durations for large specimens). We have clarified this process in the Methods section (see line 177-185). It is important to note that these additional steps were necessitated by our experimental design and resource limitations; with sufficient space or larger containers to hold an entire specimen, such rotation steps would not be required.*

#24 - Line 188 how do you know 700 grams enhances cleaning efficiency?

***Authors’ Response****:* *Sorry for the confusion. We have rewritten this section for clarity. We have also separated these two aspects in the revised text: (1) dividing large specimens among multiple containers and (2) rotating specimens between fresh containers, each stocked with larvae that had undergone a short starvation period (see lines 177-185). When the weight of the specimen exceeded that of the larvae in each container (which was the case for medium and large samples), we rotated the specimens into new containers every 6–8 hours to sustain a high level of larval feeding activity. This approach ensured that each batch of larvae was actively feeding, thereby enhancing tissue removal during each rotation cycle.*

#25 - Line 189 this adds a new variable that was not previously mentioned and runs counter to having 700 grams of larva if you are going to replace them, why not just have more larvae? Or did you mean to say within instead of between? The gray wolf was rotated every 6 hours for 78 hours? How was this time period determined?

***Authors’ Response****: We address your points in order:*

*The rotation process does not introduce a new variable, but was a practical adaptation to the limitation imposed by our container size. The maximum amount of larvae that could be added to each container was 700 grams (please see our response to comment 20); adding more would lead to overcrowding and negatively impact larval. Therefore, rather than increasing the number of larvae per container, we rotated samples into fresh containers with newly starved larvae to maintain cleaning efficiency. This approach was dictated by our experimental design and the physical limitations of the containers used. If larger containers had been available, we could have used more larvae, achieved a higher ratio, and possibly eliminated the need for rotation. This clarification has been added to the text (see line 183-185).*

*For the Gray Wolf, rotation was performed every 6–8 hours for a total cleaning duration of 78 hours, as outlined in our response to comment 23, under* ***"Timing and Rotation in Large Specimen Cleaning".***

#26 - Line 128, 194 these say the same thing

***Authors’ Response****: The repetitive section has been removed from the manuscript.*

#27 - Line 189, 195 why 6-7 hours and 6-8 hours? How do you know the Egyptian Rousette was done in one hour if it was monitored every 6-8 hours?

***Authors’ Response****: The rotation every 6–8 hours was applied only to medium and large specimens—when the sample weight was close to or exceeded the weight of the larvae in each container (please see our response to comment 23, under* ***"Timing and Rotation in Large Specimen Cleaning"****). This interval was established based on our preliminary observations to ensure that specimens were continuously exposed to active, hungry larvae, thereby maintaining cleaning efficiency. In contrast, for small specimens such as the Egyptian Rousette, cleaning was completed within one hour and did not require rotation.*

#28 - Fig 2 why did you use a picture of a species not included in this study? It also looks like B the bird has been turned over and there are significantly fewer larvae, were the larvae replaced after 6 hours?

***Authors’ Response****: We acknowledge that the specimen shown in the figure is not among those reported in this study, and we apologize for not including an image of an actual study sample. The research was performed over several years and included many specimens from various species. The image in panel A was selected because it most clearly demonstrates the cleaning process and provides higher visual quality for illustrative purposes. For panel B, the bird specimen had been turned during the cleaning process, resulting in fewer visible larvae at that moment. As per our protocol, larvae were replaced after 6 hours.*

#29 - Line 203 the f for superworms seems to be higher than dermestid beetles, as a tip try putting a wet paper towel over the specimen, it will slow down the drying process.

***Authors’ Response****: We do not have direct experience maintaining dermestid beetle colonies; therefore, we keep our comparisons between superworms and dermestids more neutral in this revised manuscript (see our responses to your related comments45). Thank you for your suggestion regarding the use of wet paper towels to slow down the drying process. While this can be effective in some cases, we have observed that superworms are capable of chewing through most soft materials, including wet paper towels, which can limit the practicality of this approach in our setup.*

#30 - Line 208 this is repetitive

***Authors’ Response****: The repetitive section has been removed from the manuscript.*

#31- Line 215 no explanation of why the bleach solution was used and bleach can damage bones.

***Authors’ Response****: In our study, we used a bleach solution for rinsing specimens in accordance with the established protocols and regulations of the museum where the work was conducted. However, as you mentioned, we are aware that bleach can potentially damage bone tissue. We have clarified in the revised manuscript that the use of bleach was required by museum protocols, which is why this step was included in our process (line 210-212). However, we now advise against using bleach to minimize the risk of bone damage.*

#32 - Line 222 if you tried those other methods it seems like you should have tried dermestids, which would be the closest comparison to superworms

***Authors’ Response****: You are correct, and we agree that a direct comparison between dermestid beetles and superworms would provide valuable information. Unfortunately, we were unable to conduct such a comparison due to lack of access to dermestid beetles and the necessary infrastructure required for their safe use. Our laboratory, which is part of the museum (ZMFUM), does not currently have the facilities or permissions needed to maintain dermestid colonies in compliance with safety protocols. Therefore, in this revised manuscript, we have kept most of our comparisons between superworms and dermestids more neutral* (*see our responses to your related comments 45).*

#33 - Line 235,6 you already established the size parameters, you can remove the entire parentheses sections here

***Authors’ Response****: it’s done.*

#34 - Line 237-239 and 248-251 say the same thing and are both also shown in the table not to mention being an obvious result, more mass takes longer.

***Authors’ Response****: The repetitive section has been removed from the manuscript.*

#35 - Line 257 I don’t think you can make this claim when none of the ratios you used fell between 10-15 your results do not clearly support this.

***Authors’ Response****:* *We acknowledge your concern. Our results demonstrated a trade-off between minimizing cleaning time and preventing damage to delicate skeletal elements. Specifically, very high larva-to-specimen ratios (such as 77.78 and 23.34 for House Mouse and Egyptian Rousette, respectively; Table 1) allowed for rapid cleaning but caused damage to fragile bones. Conversely, a lower ratio, such as 9 for Little Bittern, prevented bone damage but led to longer cleaning times. Based on these outcomes, we hypothesized that a ratio between 10 and 15 could optimize the balance between efficiency and bone preservation. To further evaluate this, we applied the 10–15 ratio to three additional bird skulls; all were thoroughly cleaned without observable bone damage (S1 Table). We have clarified this progressive, trial-based logic in the revised manuscript to better reflect the evidence and support for our conclusion.*

#36 - Line 259 You just introduced an entirely new set of specimens that were not mentioned anywhere above, and they are not comparable because you only used skulls, not the entire skeletons, how would you know if it worked for the rib bones without damage? You are also comparing damage to two mammals to birds, why did you not use mammals as a comparison? This table should also include larval biomass. In multiple places you state that 700 grams is the preferred amount of larvae but to get those ratios you would have needed to use less than 700 grams negating your claims that 700 grams is optimal. The cleaning time also significantly increased.

***Authors’ Response****: We used bird skulls in this part of the study because they were readily available as deceased specimens; no animals were killed specifically for these trials, and this allowed us to test the method across multiple samples. Additionally, we chose bird skulls because their bones are generally more fragile and vulnerable to damage than those of mammals, making them a suitable test case for assessing the gentleness of the cleaning method. While using only skulls instead of entire skeletons was not ideal, as noted in the revised manuscript, we were limited by the availability of suitable specimens and therefore were unable to test different larva-to-specimen ratios on whole skeletons (see lines 244-253). Regarding the use of 700 grams of larvae, please see the revised text (see lines 141-148) and our response to comment 20 and 25 Initially, we used 700 grams per container as this was the maximum amount that could be used given the container size in our experiment. As shown in Table 1, this led to a high larva-to-specimen ratio for small samples, which in some cases caused damage to fragile skeletons. Once the optimal ratio was identified (ratio between 10 to 15), we adjusted the amount of larvae used accordingly to prevent such damage. This process is now described more clearly in the revised manuscript, which we hope resolves the confusion. As we explain in the main text (see lines 244), there is always a trade-off between cleaning time and the risk of damage. When using a lower ratio for small samples, we observed no damage, but the cleaning process took more time.*

#37 - Line 268 where in your results can you show me that soaking accelerated the process? You would need a control to make this claim and you do not have one. Also if the tissue was so softened why did it need to get re-soaked every 12 hours?

***Authors’ Response****: We understand your concerns regarding the claim that the process of soften tissue in hot water accelerated the cleaning process and recognize the importance of proper controls. In several trials, we observed that when fresh specimens were processed without heat treatment, significant amounts of muscle and fat remained on the bones even after visible tissue decay. The presence of subcutaneous fat prevented larvae from effectively accessing underlying tissues, and the connective tissue sheaths between muscle fibers and fibrous tissues such as tendons proved particularly resistant to removal without prior heating. We already mentioned one of these examples in our response to comment 19. The wolf skull that underwent the soften process exhibited approximately a 50% reduction in cleaning time, with no observable bone damage or excessive tissue detachment. Regarding the need to repeat the soften process every 12 hours, this resulted primarily from limitations in our experimental design. Our containers could accommodate no more than 700 grams of larvae, which, as shown in Table 1, resulted in larva-to-specimen ratios for large samples that were substantially below the optimal ratio range (often less than 3, whereas 10–15 is optimal). With these lower ratios, larvae could not remove tissues as quickly, sometimes necessitating to repeat this softening process in hot water to soften remaining tissues and facilitate cleaning. If larger containers and increased larval biomass had been available to allow for optimal ratios, we believe that cleaning would have proceeded much faster and likely eliminated the need for repeated softening steps.*

#38 - Line 269 what internal areas? You already removed all the skin, excess tissue and organs

***Authors’ Response****: This refers to internal spaces such as the cranial cavity (inside the skull), vertebral foramina, and other small cavities within bones that are not accessible by simple skinning or manual dissection.*

#39 - Line 268-271 you said the same thing twice

***Authors’ Response****: We have removed the redundant part.*

#40 - Line 275 fig 4 is not a close enough picture to see any type of damage to the bones, especially if they have already been covered in a varnish

***Authors’ Response****: Figure 4 was intended to present multiple skeletal samples in a single frame for general overview. To allow for closer inspection, we have provided individual high-resolution images of each specimen as supplementary material (*S3 Fig_S7 Fig*).*

#41 - Line 291 what facilities are using chemical treatments?

***Authors’ Response:*** *We appreciate your comment. To be more accurate, we have revised the text to state that chemical treatments have been used in some facilities, rather than stating they remain prevalent. We have also removed the phrase “remain prevalent” to avoid suggesting universal practice (see lines 261-262).*

#42 - Line 306 local to where? This is an international publication your local and your readers local are likely not the same

***Authors’ Response****: We have revised this part of the manuscript and now only mention the widespread use of superworms in the pet and feed industries*.

#43 - Line 315 your citation number is wrong (unless it’s a tracked changes issue)

***Authors’ Response****: This was due to tracked changes, and we have double-checked and corrected the citation numbering throughout the revised manuscript.*

#44 - Line 318-9 you say they don’t fly then you say the can fly, also missing a citation

***Authors’ Response:*** *We apologize for the confusion. Adult beetles are capable of very weak and rare flight. Based on our observations during several years of superworm breeding, such flight attempts occur only under extreme stress. This has now been clarified, and the relevant reference has been added in the revised manuscript (see lines 276-278).*

#45 - Line 345/table 2 none of the animals listed in that table are the same as the animals you used. There is no indication of how those specimens were prepared, they could have been complete animals with skin/fur/organs still present. No indication of how many dermestids were used. You do not have the evidence to support your claim that “superworms demonstrated a markedly shorter cleaning duration compared to dermestid beetles.”

***Authors’ Response****:* *We totally agree that we cannot directly compare our results with those from the studies listed in Table 2. The specimens referenced, which were cleaned by dermestid beetles, are drawn from various studies and do not directly match the species, specimen preparation, or cleaning protocols used in our work. As you noted, important methodological details such as dermestid pre-conditioning (e.g., starving, regular replacement) are not stated, making the cleaning durations difficult to compare. Therefore, we have removed all statements comparing the cleaning speed of beetles and superworms from the manuscript. We now only note that our results suggest superworms have potential as a cleaning agent under our specific protocol, and we have refrained from making direct efficacy claims due to the lack of comparable data.*

#46 - Line 347 saying “beetle-based cleaning often requires several weeks is deceptive, the human jawbone is clearly an outlier and the only other one was an entire deer which is much larger than your large category. Your “large” is not the same as large, a clean cow skull by itself could weigh 3-4kg.

***Authors’ Response****: Please see our response to comment 45. We acknowledge your point that the comparison is not appropriate due to differences in specimen size and type. We have removed any misleading statements about the duration of beetle-based cleaning from the manuscript.*

#47 - Line 357 not supported by your results

***Authors’ Response:*** *Please refer to our response to comment 35, where we address this concern in detail.*

#48 - Line 360 700 grams of larvae were not used for your extra owl skulls.

***Authors’ Response****: As described in the Methods section (see line 140), in the first part we aimed to determine the optimal larva-to-specimen ratio. For this reason, we used fixed-size containers for all samples and filled each container with 700 grams of larvae, as this is the maximum amount that can be accommodated in these containers without causing overcrowding or negatively affecting larval performance (see also our response to comment 20). After these trials, we identified the optimal ratio that balances cleaning time and the risk of damage to fragile bones (lines 306-309). Subsequently, when using the extra owl skulls, we adjusted the number of larvae according to the specimen’s weight to achieve the optimal ratio (10–15). This allowed us to test whether maintaining this ratio could prevent the type of damage to fragile bones observed in smaller specimens in the initial part of our study—where using the fixed amount of 700 grams per container resulted in very high larva-to-specimen ratios (see Table 1 for Egyptian Rousette and House Mouse). This process and its rationale are now clearly described in the revised text (see lines 202-206).*

#49 - Line 364-5 you are doing things that you can’t make a clear comparison to dermestid beetles, did any of the specimens listed in table 2 use starving dermestids or switch them out every 6-8 hours?

***Authors’ Response****: We fully agree that we cannot directly compare our results with those from the studies listed in Table 2, as discussed in our response to comment 45. The available studies do not specify whether starving dermestids or regular replacement was used, making a clear comparison impossible. We have revised the manuscript to address these limitations and removed any direct performance comparisons between dermestid beetles and superworms.*

#50 - Line 366 again, you do not have a control that proves that repositioning made a difference

***Authors’ Response****: During our trials, we qualitatively observed that repositioning specimens seemed to improve cleaning efficiency, likely by ensuring more continuous exposure to active, hungry larvae. However, we agree that we did not include a control to directly test this effect, and further controlled studies are needed to confirm it. In the revised manuscript, we have presented this as a suggestion based on our observations rather than a definitive conclusion.*

#51 - Line 372 this is the first mention of odor

***Authors’ Response:*** *This is not a new variable; our intention was simply to suggest performing the experiment in a well-ventilated area. We apologize for any confusion caused by the wording, and this point has been clarified in the revised version (see lines 314–315).*

#52 - Line 375 missing citation, who recommends?

***Authors’ Response:*** *It’s added.*

#53 - Line 377 Citation number is wrong

***Authors’ Response:*** *It’s corrected*

**Reviewer 2**

I believe that this revision of the manuscript is more comprehensive in terms of its methods and intentions. The cleaning of osteological material has been the subject of various studies, so having an alternative to chemical methods and dermestid beetle cultures is extremely helpful, especially in countries where it is difficult to maintain laboratories with the appropriate conditions for handling chemicals or where dermestid beetles are difficult to obtain. The publication of these results in this journal is important given its international reach, offering an alternative or coexistence with other chemical or biological methods of cleaning bone material.

***Authors’ Response****: We sincerely appreciate your positive feedback and your recognition of the comprehensive revisions made to our manuscript. We agree that offering practical alternatives for skeletal cleaning is especially valuable in diverse international contexts. Thank you for highlighting the importance of our results and for supporting publication in this journal.*
