# Supplementary material for: A practical and safe alternative method for skeletal cleaning for museum specimens using superworms (Zophobas morio)
Source: PLoS One. 2026 Jul 1;21(7):e0349669. doi: 10.1371/journal.pone.0349669 (PMC13322520; doi:10.1371/journal.pone.0349669)
Supplement: Peer review history

# Peer review: Decision Letter 0

**DOI:** 10.1371/journal.pone.0349669.r001

**Authors:** Wesley D. Colombo

11 Nov 2025

PONE-D-25-57203

Efficient skeletal cleaning using superworms (*Zophobas morio* larvae): a practical alternative method for museum specimens

PLOS ONE

Dear Dr. Monfared,

Thank you for submitting your manuscript to PLOS ONE. After careful consideration, we feel that it has merit but does not fully meet PLOS ONE’s publication criteria as it currently stands. Therefore, we invite you to submit a revised version of the manuscript that addresses the points raised during the review process.

====================================================

<article class="text-token-text-primary w-full focus:outline-none [--shadow-height:45px] has-data-writing-block:pointer-events-none has-data-writing-block:-mt-(--shadow-height) has-data-writing-block:pt-(--shadow-height) [&:has([data-writing-block])>*]:pointer-events-auto [content-visibility:auto] supports-[content-visibility:auto]:[contain-intrinsic-size:auto_100lvh] scroll-mt-[calc(var(--header-height)+min(200px,max(70px,20svh)))]" data-scroll-anchor="true" data-testid="conversation-turn-8" data-turn="assistant" data-turn-id="request-WEB:22a205ca-4713-4909-a4eb-a136b1b93080-3" dir="auto" tabindex="-1">

The manuscript presents a valuable and creative contribution, proposing the use of *Zophobas morio* larvae as an alternative method for cleaning skeletal material in scientific collections. The approach is relevant and promising, particularly for medium and large specimens, but several essential revisions are needed before the paper can be considered for publication.

The main mandatory changes concern the **clarity, consistency, and justification of methods and results**. The **ratios of larvae to specimen mass** are inconsistent between the abstract, figures, tables, and text, and must be standardized throughout. The **starvation period** for larvae should be supported by experimental evidence or properly referenced; otherwise, the claim that it improves cleaning efficiency should be removed or rephrased. The **boiling process** also requires a clear rationale and description, as it poses potential risks to bone integrity. If maintained, the authors should explain why boiling was chosen, how its duration was determined, and why alternatives such as soaking were not preferred. The **description of specimen weights** should clarify whether values refer to intact or partially processed specimens, and repeated or redundant information should be reduced.

In the **results and discussion**, several statements need revision or better support. The claim that *Z. morio* is less damaging than dermestid beetles conflicts with observations of bone damage within twelve hours. Assertions about reduced costs, faster processing, or operational efficiency are not supported by data and should either be substantiated or removed. Many passages require appropriate citations, and a few references are inaccurate or unrelated to the arguments made. The section introducing the marbled polecat and its cleaning method should appear earlier, preferably within the methodology.

The **title** should also reflect the actual scope of the work, emphasizing that the technique is particularly effective for medium and large specimens. The reviewers suggest a revised title such as “Efficient skeletal cleaning using superworms (*Zophobas morio* larvae): a practical alternative method for medium and large museum specimens.”

Finally, attention to **language and style** is important: minor errors (e.g., “exciting” instead of “existing”) should be corrected, vague expressions clarified, and repetition of Latin names avoided. Consistent terminology for specimen sizes (small, medium, large) is recommended.

In summary, the study is scientifically sound and potentially valuable, but a **major revision** is required. The authors must focus on standardizing methodological details, justifying experimental choices, removing unsupported claims, and ensuring all statements are properly cited. With these corrections, the manuscript could make a strong and original contribution to the literature on skeletal cleaning methods.

====================================================

Please submit your revised manuscript by Dec 26 2025 11:59PM. If you will need more time than this to complete your revisions, please reply to this message or contact the journal office at plosone@plos.org. When you're ready to submit your revision, log on to https://www.editorialmanager.com/pone/ and select the 'Submissions Needing Revision' folder to locate your manuscript file.

</article>

Please include the following items when submitting your revised manuscript:

- A rebuttal letter that responds to each point raised by the academic editor and reviewer(s). You should upload this letter as a separate file labeled 'Response to Reviewers'.
- A marked-up copy of your manuscript that highlights changes made to the original version. You should upload this as a separate file labeled 'Revised Manuscript with Track Changes'.
- An unmarked version of your revised paper without tracked changes. You should upload this as a separate file labeled 'Manuscript'.

If you would like to make changes to your financial disclosure, please include your updated statement in your cover letter. Guidelines for resubmitting your figure files are available below the reviewer comments at the end of this letter.

If applicable, we recommend that you deposit your laboratory protocols in protocols.io to enhance the reproducibility of your results. Protocols.io assigns your protocol its own identifier (DOI) so that it can be cited independently in the future. For instructions see: https://journals.plos.org/plosone/s/submission-guidelines#loc-laboratory-protocols. Additionally, PLOS ONE offers an option for publishing peer-reviewed Lab Protocol articles, which describe protocols hosted on protocols.io. Read more information on sharing protocols at https://plos.org/protocols?utm_medium=editorial-email&utm_source=authorletters&utm_campaign=protocols.

We look forward to receiving your revised manuscript.

Kind regards,

Wesley D. Colombo

Academic Editor

PLOS ONE

Journal Requirements:

When submitting your revision, we need you to address these additional requirements.

1. Please ensure that your manuscript meets PLOS ONE's style requirements, including those for file naming. The PLOS ONE style templates can be found at https://journals.plos.org/plosone/s/file?id=wjVg/PLOSOne_formatting_sample_main_body.pdf and https://journals.plos.org/plosone/s/file?id=ba62/PLOSOne_formatting_sample_title_authors_affiliations.pdf

2. Thank you for stating the following in your Competing Interests section:

No

Please complete your Competing Interests on the online submission form to state any Competing Interests. If you have no competing interests, please state "The authors have declared that no competing interests exist.", as detailed online in our guide for authors at http://journals.plos.org/plosone/s/submit-now

This information should be included in your cover letter; we will change the online submission form on your behalf.

3. We note that Figure(s) 1, 2, 4, S1 and S2 in your submission contain copyrighted images. All PLOS content is published under the Creative Commons Attribution License (CC BY 4.0), which means that the manuscript, images, and Supporting Information files will be freely available online, and any third party is permitted to access, download, copy, distribute, and use these materials in any way, even commercially, with proper attribution. For more information, see our copyright guidelines: http://journals.plos.org/plosone/s/licenses-and-copyright.

We require you to either (1) present written permission from the copyright holder to publish these figures specifically under the CC BY 4.0 license, or (2) remove the figures from your submission:

a. You may seek permission from the original copyright holder of Figure(s) 1, 2, 4, S1 and S2 to publish the content specifically under the CC BY 4.0 license.

We recommend that you contact the original copyright holder with the Content Permission Form (http://journals.plos.org/plosone/s/file?id=7c09/content-permission-form.pdf) and the following text:

“I request permission for the open-access journal PLOS ONE to publish XXX under the Creative Commons Attribution License (CCAL) CC BY 4.0 (http://creativecommons.org/licenses/by/4.0/). Please be aware that this license allows unrestricted use and distribution, even commercially, by third parties. Please reply and provide explicit written permission to publish XXX under a CC BY license and complete the attached form.”

Please upload the completed Content Permission Form or other proof of granted permissions as an "Other" file with your submission.

In the figure caption of the copyrighted figure, please include the following text: “Reprinted from [ref] under a CC BY license, with permission from [name of publisher], original copyright [original copyright year].”

b. If you are unable to obtain permission from the original copyright holder to publish these figures under the CC BY 4.0 license or if the copyright holder’s requirements are incompatible with the CC BY 4.0 license, please either i) remove the figure or ii) supply a replacement figure that complies with the CC BY 4.0 license. Please check copyright information on all replacement figures and update the figure caption with source information. If applicable, please specify in the figure caption text when a figure is similar but not identical to the original image and is therefore for illustrative purposes only.

4. Please upload a copy of Supporting Information Table S1, which you refer to in your text on page 8 and 9.

5. We notice that your supplementary [table] are included in the manuscript file. Please remove them and upload them with the file type 'Supporting Information'. Please ensure that each Supporting Information file has a legend listed in the manuscript after the references list.

6. If the reviewer comments include a recommendation to cite specific previously published works, please review and evaluate these publications to determine whether they are relevant and should be cited. There is no requirement to cite these works unless the editor has indicated otherwise.

[Note: HTML markup is below. Please do not edit.]

Reviewers' comments:

Reviewer's Responses to Questions

**Comments to the Author**

1. Is the manuscript technically sound, and do the data support the conclusions?

The manuscript must describe a technically sound piece of scientific research with data that supports the conclusions. Experiments must have been conducted rigorously, with appropriate controls, replication, and sample sizes. The conclusions must be drawn appropriately based on the data presented.

Reviewer #1: Partly

Reviewer #2: Yes

**********

2. Has the statistical analysis been performed appropriately and rigorously?

Reviewer #1: N/A

Reviewer #2: N/A

**********

3. Have the authors made all data underlying the findings in their manuscript fully available?

The PLOS Data policy requires authors to make all data underlying the findings described in their manuscript fully available without restriction, with rare exception (please refer to the Data Availability Statement in the manuscript PDF file). The data should be provided as part of the manuscript or its supporting information, or deposited to a public repository. For example, in addition to summary statistics, the data points behind means, medians and variance measures should be available. If there are restrictions on publicly sharing data—e.g. participant privacy or use of data from a third party—those must be specified.

Reviewer #1: Yes

Reviewer #2: Yes

**********

4. Is the manuscript presented in an intelligible fashion and written in standard English?

PLOS ONE does not copyedit accepted manuscripts, so the language in submitted articles must be clear, correct, and unambiguous. Any typographical or grammatical errors should be corrected at revision, so please note any specific errors here.

Reviewer #1: Yes

Reviewer #2: Yes

**********

5. Review Comments to the Author

Please use the space provided to explain your answers to the questions above. You may also include additional comments for the author, including concerns about dual publication, research ethics, or publication ethics. (Please upload your review as an attachment if it exceeds 20,000 characters)

Reviewer #1: Line 57 vertebrates or vertebrate species

Line 64 “a long time” add some more detail – decades, over 100 years

Line 77 “exciting” should be existing

Line 78 (and others) you already gave the Latin name, you do not need to keep providing it.

The ratios that you use for larva-to-specimens is confusing, inconsistent, and not in the right format. These sections need to be rewritten. Figure 1 says 7:1 but the abstract says 77.78:1 and line 140 says 10 to 70. Line 151 is wrong and the ratios don’t match table 1.

Line 112 were the super worms removed from the wheat bran to starve them?

Line 114 “this short starvation period (appeared to) stimulate the larvae”

Line 115 what study or example are you using to claim significantly improved efficiency. Did you test with and without having a day of starvation? You do not know they were ravenous.

Line 117 and 102 are essentially identical, the same sentence stating we used eight different specimens shows up in lines 28, 91, 102, 117, 175. Cut out some of the repetition.

Line 119 are the weights listed for intact specimens or are they the weights after skin and tissue removal? The groups can just be small, medium, large, you can cut “-sized specimens”.

Line 131 boiling a specimen, which could be considered a form of maceration, has the potential to damage bones. What it the justification for boiling these specimens, why are the risks not addressed, and how was the length of time determined? If boiling was used to soften tissue, why did you not just soak it in water like you did with the mummified bat, or conversely boil the bat? A properly skinned and cleaned Eurasian Eagle Owl should not have a significant amount of flesh remaining.

Line 138 samples would imply that you only put part of the specimen in the container, is that true?

Line 145 Wouldn’t the wheat bran serve as an additional source of food, which would run counter to the rotation to keep them hungry and active?

Line 146 if the specimens were “fully submerged” why did they need to be repositioned and how could you check the process? The specimen in Figure 2 is not fully submerged.

Line 175-177 this is not a result, delete

Line 189 is not worded correctly, you added the larval biomas so “indicating that larger species require longer cleaning times and have greater larval biomass” doesn’t make sense. Possibly require larger biomas?

Line 191 is redundant, just use small specimens you don’t need to list the species as they were already established

Line 192 in your abstract and introduction (lines 23-27, 37, 65) you claim that superworms are better and safer but here you are saying they are damaging the bones and that damage is happening within 12 hours. That does not seem safer or better than dermestids. Line 72 “Dermestid beetles efficiently remove soft tissues while preserving the delicate bone structures”

Line 198 where is your evidence that boiling “accelerated the process”

Line 199 boiling for that duration wouldn’t cause visible damage but it can cause long term damage

Line 203 “providing an advantage over conventional methods that often require drilling or sawing” This needs a citation and as far as I know is not a standard practice for any of the other cleaning methods you listed.

Line 214 this is a comment, not a needed change but for improved scanning accuracy you should not be covering the specimen in a varnish.

Line 214-15 this needs a citation

Line 218-219 the bones were damaged using superworms, operating costs were not addressed anywhere in the manuscript and your method also includes both manual dissection and maceration steps. This claim is not supported.

Line 221 why is this the first mention of the marbled Polecat and the alternate method used? This should have been addressed much earlier in the manuscript.

Line 224 missing citation.

Line 228 the challenges are not “substantial” as evidenced by all the institutions using that method on line 70. The citation used explains how to safely contain dermestid beetles.

Line 231 the citation used does not say this, in fact, it essentially says the exact opposite. This is also not true unless you are speaking about suppliers local to you, in which case that needs to be clarified.

Line 242 but dermestids can feed as adults, meaning their feeding period does not end.

Line 250 cost is not listed anywhere in this manuscript and how are you assessing these costs? Letting a specimen sit in dermestid beetles for slightly longer shouldn’t add additional costs.

Line 256-258 you can feed dermestid beetles (or superworms) tissue removed from specimens, reducing the cost of food to 0.

Line 252 there are no actual costs listed so how does the reader know what the costs are? Colony maintenance is not addressed in the manuscript, so how is the colony being maintained, are you buying new superworms every 10-12 weeks?

Line 262, 264 missing citation

Line 266 this seems like it should have been mentioned much earlier in the manuscript and like an issue for an institution using this method. Dermestids do not require diet supplementation.

Line 276 cost was not proven

Line 280 this is the first mention of mealworms in the entire manuscript.

Line 385 this only illustrates a risk for boiling, not for “traditional defleshing techniques”

Are new worms being purchased regularly? Monitoring every 6-8 hours and repositioning seems like more work than using other methods (line 148) tissue getting to dry after 12-16 hours is not a problem for dermestids (155) line 39 contamination risk is not addressed

Reviewer #2: This manuscript clearly shows a viable option for cleaning bone material in scientific collections, in which the bone characteristics of the specimens must be kept as complete as possible.

In this regard, the method using the larvae of the beetle Tenebrionidae Zophobas morio meets the desired characteristics for collections: it is efficient, takes as little time as possible, and causes the least damage to bone structures; it also reduces contaminating waste and has a low potential to become a pest in the collection. However, as the authors point out, the method is particularly efficient for medium to large specimens, so perhaps this detail could be reflected in the title “Efficient skeletal cleaning using superworms (Zophobas morio larvae): a practical alternative method for medium and large museum specimens.”

In the same vein, it would be useful to compare the effectiveness of the larvae of the other species mentioned for smaller specimens against the larvae of the early stages of Z. morio to try to avoid the establishment of colonies of various species of darkling beetles.

On the other hand, it is suggested that the authors seek less aggressive alternatives to sodium hypochlorite for bleaching specimens, as it has been documented that if it is not completely neutralized, it continues to act on the mineral matrix of the bone.

**********

6. PLOS authors have the option to publish the peer review history of their article (what does this mean?). If published, this will include your full peer review and any attached files.

If you choose “no”, your identity will remain anonymous but your review may still be made public.

**Do you want your identity to be public for this peer review?** For information about this choice, including consent withdrawal, please see our Privacy Policy.

Reviewer #1: No

Reviewer #2: No

**********

[NOTE: If reviewer comments were submitted as an attachment file, they will be attached to this email and accessible via the submission site. Please log into your account, locate the manuscript record, and check for the action link "View Attachments". If this link does not appear, there are no attachment files.]

To ensure your figures meet our technical requirements, please review our figure guidelines: https://journals.plos.org/plosone/s/figures

You may also use PLOS’s free figure tool, NAAS, to help you prepare publication quality figures: https://journals.plos.org/plosone/s/figures#loc-tools-for-figure-preparation.

NAAS will assess whether your figures meet our technical requirements by comparing each figure against our figure specifications.

# Author comment: Author response to Decision Letter 1

**DOI:** 10.1371/journal.pone.0349669.r002

6 Jan 2026

Dear Editor, dear Reviewers,

We are pleased to resubmit a revised version of our manuscript, “A practical and safe alternative method for skeletal cleaning of museum specimens using superworms (Zophobas morio)”. We sincerely thank the reviewers for their positive assessment and constructive comments. We have addressed and responded to all comments, as detailed below.

Notably, we rewrote the section of the manuscript concerning the larva to specimen weight ratio, which we believe caused significant confusion in the original submission. By revising this section, we believe we have addressed the major concerns raised by the Editor and Reviewer 1. We hope that the Editor and Reviewers will find the revised manuscript suitable for publication in PLOS One.

With kind regards, on behalf of the authors,

Morteza Monfared

RESPONSES TO AE AND REVIEWER COMMENTS

Editor Comments

#1 - The ratios of larvae to specimen mass are inconsistent between the abstract, figures, tables, and text, and must be standardized throughout

Authors’ Response: We agree that the original presentation of these data was confusing and lacked a consistent format throughout the manuscript. This issue was also pointed out by the first reviewer. In the revised version, we have thoroughly reworked this section to improve clarity and consistency. Specifically, we have revised the description of the larva-to-specimen ratio in the Methods (lines 146–150) and Results (lines 224–234) sections. We have also modified Table 1 to clarify this information and added new results obtained using the optimal ratio to the Supplementary Material as Table S1.

#2 - The starvation period for larvae should be supported by experimental evidence or properly referenced; otherwise, the claim that it improves cleaning efficiency should be removed or rephrased.

Authors’ Response: Based on our personal experience, this short starvation period noticeably affects the larvae’s cleaning performance. However, since we do not have data to support this here, we have rephrased this part to indicate that we used this approach and propose it as a suggestion, please see the lines 131-132.

#3 - The boiling process also requires a clear rationale and description, as it poses potential risks to bone integrity. If maintained, the authors should explain why boiling was chosen, how its duration was determined, and why alternatives such as soaking were not preferred.

Authors’ Response: We have already addressed this comment in the revised manuscript. To clarify, by “boiling” we did not mean the traditional boiling step. Instead, we soaked the samples in hot water at temperatures up to 80°C to gently soften the tissues and accelerate the cleaning process. To better reflect this procedure and avoid confusion, we have replaced the term “boiling” with “soaking” throughout the text.

#4 - The description of specimen weights should clarify whether values refer to intact or partially processed specimens, and repeated or redundant information should be reduced.

Authors’ Response: These weights refer to the specimens after skinning and removal of soft tissues. We added to the text, please see line 127-128.

#5 - In the results and discussion, several statements need revision or better support. The claim that Z. morio is less damaging than dermestids beetle conflicts with observations of bone damage within twelve hours. Assertions about reduced costs, faster processing, or operational efficiency are not supported by data and should either be substantiated or removed.

Authors’ Response: We rewrote most of the Discussion section based on the comments from the first reviewer. In the revised version, we now explain more clearly the advantages of using superworms in terms of speed and operational efficiency. We also removed the parts related to cost, as in our experimental setup we could not directly compare the costs of the two approaches.

#6 - Many passages require appropriate citations, and a few references are inaccurate or unrelated to the arguments made. The section introducing the marbled polecat and its cleaning method should appear earlier, preferably within the methodology.

Authors’ Response: regarding the reference part, we worked on it and we added more appropriate references and regarding the part of the marbled polecat, thanks for pointing this out, we move this part into the method (lines 101-105) and result part (lines 200- 204).

#7 - The title should also reflect the actual scope of the work, emphasizing that the technique is particularly effective for medium and large specimens. The reviewers suggest a revised title such as “Efficient skeletal cleaning using superworms (Zophobas morio larvae): a practical alternative method for medium and large museum specimens.”

Authors’ Response: We have changed the title based on the revised version of the manuscript and the identification of the optimal ratio, under which no damage to delicate bones was observed. Please see the description of the optimal ratio in the Methods (lines 146–150), Results (lines 224–234), and Discussion (lines 292–298) sections.

Reviewer 1

#1 - Line 57 vertebrates or vertebrate species

Authors’ Response: It’s done.

#2 - Line 64 “a long time” add some more detail – decades, over 100 years

Authors’ Response: It’s done.

#3 - Line 77 “exciting” should be existing

Authors’ Response: It’s done.

#4 - Line 78 (and others) you already gave the Latin name, you do not need to keep providing it.

Authors’ Response: It’s done.

#5 - The ratios that you use for larva-to-specimens is confusing, inconsistent, and not in the right format. These sections need to be rewritten. Figure 1 says 7:1 but the abstract says 77.78:1 and line 140 says 10 to 70. Line 151 is wrong and the ratios don’t match table 1.

Authors’ Response: Thank you for pointing out the inconsistencies regarding the larva to specimen ratios. We agree that the original presentation of these data was confusing and did not maintain a consistent format throughout the manuscript. In this revised version, we have thoroughly reworked this section for clarity and consistency.

Specifically, we have revised the description of larva to specimen ratio in the Methods (lines 146–150) and Result (lines 224–234) section. We also modify the Table 1 to make this part more clear and we added the new result using the optimal ratio in the supplementary as table S1.

#6 - Line 112 were the super worms removed from the wheat bran to starve them?

Authors’ Response: The wheat bran used as a substrate for the superworms was not fresh but had been recycled multiple times through previous rearing cycles. Consequently, its nutritional content was substantially reduced and it could not effectively serve as a food source for the larvae. We therefore used this bran primarily as a holding substrate, which also helped reduce humidity around the larvae. For the purposes of this study, the larvae were considered to be in a state of starvation for at least 24 hours before being placed in the cleaning containers, ensuring that they were highly active and motivated to consume the soft tissues of the specimens. This information has been added to the manuscript in lines 112–114. Moreover, we rephrased this section to present it as a recommendation for increasing the efficiency of superworms in skeletal cleaning.

#7 - Line 114 “this short starvation period (appeared to) stimulate the larvae”

Authors’ Response: It’s added.

#8 - Line 115 what study or example are you using to claim significantly improved efficiency. Did you test with and without having a day of starvation? You do not know they were ravenous.

Authors’ Response: Based on our observations during the experiments, we found that a brief starvation period of approximately 24 hours noticeably increased larval activity, thereby enhancing both the speed and efficiency of the cleaning process. This short starvation period ensured that all larvae were actively engaged in cleaning when introduced to the samples. Based on our personal experience, larvae were therefore starved for 24 hours prior to use to stimulate feeding activity and increase their effectiveness in soft tissue removal. This point has been included in the manuscript as a recommendation based on our observations (lines 131–132).

#9 - Line 117 and 102 are essentially identical, the same sentence stating we used eight different specimens shows up in lines 28, 91, 102, 117, 175. Cut out some of the repetition.

Authors’ Response: The repitations were removed

#10 - Line 119 are the weights listed for intact specimens or are they the weights after skin and tissue removal? The groups can just be small, medium, large, you can cut “-sized specimens”.

Authors’ Response: Yes, the weights reported in the manuscript refer to specimens after skin removal for all samples and after soft tissue removal for large samples. This clarification has been added to the manuscript in line 127.

#11 - Line 131 boiling a specimen, which could be considered a form of maceration, has the potential to damage bones. What it the justification for boiling these specimens, why are the risks not addressed, and how was the length of time determined? If boiling was used to soften tissue, why did you not just soak it in water like you did with the mummified bat, or conversely boil the bat? A properly skinned and cleaned Eurasian Eagle Owl should not have a significant amount of flesh remaining.

Authors’ Response: we totally agree with you and in the manuscript, by “boiling” we did not mean the boiling step used in the traditional method. Instead, the samples were soaked in hot water (at temperatures up to 80 °C) solely to soften the tissues and accelerate the cleaning process. To better reflect this procedure, we have replaced the term “boiling” with “soaking” in the manuscript.

#12 - Line 138 samples would imply that you only put part of the specimen in the container, is that true?

Authors’ Response: For large specimens only, we divided the samples into several smaller pieces so that they could fit into the containers, as the same type of container was used for all specimens.

#13 - Line 145 Wouldn’t the wheat bran serve as an additional source of food, which would run counter to the rotation to keep them hungry and active?

Authors’ Response: As we explained in the comments 6, we used the not fresh bran that could not effectively serve as a food source for the larvae.

#14 - Line 146 if the specimens were “fully submerged” why did they need to be repositioned and how could you check the process? The specimen in Figure 2 is not fully submerged

Authors’ Response: Yes, the small samples were fully submerged, whereas this was not the case for the medium and large samples due to the size of the containers. We have revised the text accordingly and now use the term “submerged” instead of “fully submerged.”

#15 - Line 175-177 this is not a result, degree delete

Authors’ Response: It’s done.

#16 - Line 189 is not worded correctly, you added the larval biomas so “indicating that larger species require longer cleaning times and have greater larval biomass” doesn’t make sense. Possibly require larger biomas?

Authors’ Response: Yes, it is the larger biomass. Thank you for pointing this out; this has been corrected in the manuscript.

#17 - Line 191 is redundant, just use small specimens you don’t need to list the species as they were already established

Authors’ Response: It’s done.

#18 - Line 192 in your abstract and introduction (lines 23-27, 37, 65) you claim that superworms are better and safer but here you are saying they are damaging the bones and that damage is happening within 12 hours. That does not seem safer or better than dermestids. Line 72 “Dermestid beetles efficiently remove soft tissues while preserving the delicate bone structures”

Authors’ Response: We have corrected this part in the text. Indeed, damage occurred in the small specimens when suboptimal conditions were used. By applying the optimal larva-to-specimen ratio, we overcame this issue, and no damage was observed in the small skulls tested when using a ratio between 10 and 15.

#19 - Line 198 where is your evidence that boiling “accelerated the process”

Authors’ Response: Based on our experience during the experiments, we compared the cleaning process for large and medium-sized samples with and without soaking in hot water. For example, in the case of a hyena skull, we found that pre-soaking significantly accelerated the cleaning process. Specifically, soaking reduced the cleaning time by approximately 40%, indicating that this step can substantially improve efficiency.

#20 - Line 199 boiling for that duration wouldn’t cause visible damage but it can cause long term damage

Authors’ Response: We fully agree with this point, and one of the advantages of our approach compared to the traditional boiling method is that it does not involve actual boiling. As explained in responses to Comments 6 and 7, we only soaked the samples in hot water, a step that did not cause any damage to the specimens.

#21 - Line 203 “providing an advantage over conventional methods that often require drilling or sawing” This needs a citation and as far as I know is not a standard practice for any of the other cleaning methods you listed.

Authors’ Response: In some museums, for example the Museum of FUM, we have observed such cases. However, because we could not find suitable references to support this statement, we have removed this part from the manuscript.

#22 - Line 214 this is a comment, not a needed change but for improved scanning accuracy you should not be covering the specimen in a varnish.

Authors’ Response: Thanks for your comments, it has been added.

#23 - Line 214-15 this needs a citation

Authors’ Response: It’s added.

#24 - Line 218-219 the bones were damaged using superworms, operating costs were not addressed anywhere in the manuscript and your method also includes both manual dissection and maceration steps. This claim is not supported.

Authors’ Response: This part is removed in the new revised version.

#25 - Line 221 why is this the first mention of the marbled Polecat and the alternate method used? This should have been addressed much earlier in the manuscript.

Authors’ Response: Yes, you are correct. We have added this information to the beginning of the Methods section.

#26 - Line 224 missing citation.

Authors’ Response: It’s added.

#27 - Line 228 the challenges are not “substantial” as evidenced by all the institutions using that method on line 70. The citation used explains how to safely contain dermestid beetles.

Authors’ Response: Thanks for pointing this out, it is corrected in the new version.

#28 - Line 231 the citation used does not say this, in fact, it essentially says the exact opposite. This is also not true unless you are speaking about suppliers local to you, in which case that needs to be clarified.

Authors’ Response: This error occurred during the referencing process, and we apologize for it. In this updated version, we have carefully reviewed the references and corrected the mistake.

#29 - Line 242 but dermestids can feed as adults, meaning their feeding period does not end.

Authors’ Response: That is correct; however, feeding activity during the larval stage is more effective compared to other stages. In our study, we used only the larval stage of the superworms, resulting in higher feeding efficiency compared to beetles, where both larvae and adults are involved. An additional advantage is that managing superworms, all at the same larval stage, is much easier and reduces the risk of contamination to museum collections.

#30 - Line 250 cost is not listed anywhere in this manuscript and how are you assessing these costs? Letting a specimen sit in dermestid beetles for slightly longer shouldn’t add additional costs.

Authors’ Response: We agree that it is difficult to directly assess the cost of the experiment. By “cost-effective,” we intended to convey that this approach is faster, as all the specimens are in the larval stage, making colony management easier and safer. It does not require post-processing steps after cleaning, nor does it involve removing eggs, which is particularly important for the safety of museum samples. Overall, this method is le

Attachment Submitted filename: Response.docx

# Peer review: Decision Letter 1

**DOI:** 10.1371/journal.pone.0349669.r003

**Authors:** Wesley D. Colombo

8 Feb 2026

PONE-D-25-57203R1

A practical and safe alternative method for skeletal cleaning for museum specimens using superworms (Zophobas morio)

PLOS One

Dear Dr. Monfared,

Thank you for submitting your manuscript to PLOS ONE. After careful consideration, we feel that it has merit but does not fully meet PLOS ONE’s publication criteria as it currently stands. Therefore, we invite you to submit a revised version of the manuscript that addresses the points raised during the review process.

Based on Reviewer 1’s comments, the manuscript requires substantial revision before it can be reconsidered for publication. The review identifies significant shortcomings in methodological rigor, clarity, and evidentiary support, which currently undermine several of the manuscript’s main conclusions.

A central concern is the use of misleading terminology. Referring to water at 80 °C as “soaking” is inappropriate and must be corrected. The procedure should be described precisely, including how temperature was measured, whether it was maintained or allowed to cool, and the exact duration of exposure.

Several key conclusions are not supported by the experimental design. Claims that soaking accelerated cleaning or that repositioning specimens improved efficiency lack proper controls and therefore must be removed or clearly rephrased as descriptive observations rather than causal statements.

The comparison with dermestid beetles is also problematic. The manuscript does not provide comparable data to support claims of superior cleaning speed, and Table 2 includes inconsistent specimens and questionable citations. This comparison should be removed, substantially revised, or strongly qualified.

Methodological details are insufficient in multiple areas, including larval replacement, specimen repositioning, number of containers used for large specimens, monitoring intervals, and criteria for determining completion. These variables must be clearly defined to ensure reproducibility.

In addition, several numerical claims, particularly regarding the optimal use of 700 g of larvae and monthly larval production, are not adequately supported and appear inconsistent. These statements require justification or removal.

The manuscript also contains redundancy, unclear statements, inconsistent terminology (e.g., “samples” vs. “specimens”), and errors in scientific naming and citation. Tables and figures need revision to provide meaningful quantitative information and visual evidence that supports the claims made.

In summary, major revision is required. The authors must address methodological gaps, remove or qualify unsupported claims, improve clarity and consistency, and correct all issues related to data presentation and citations before the manuscript can be reconsidered for publication.

Please submit your revised manuscript by Mar 25 2026 11:59PM. If you will need more time than this to complete your revisions, please reply to this message or contact the journal office at plosone@plos.org. When you're ready to submit your revision, log on to https://www.editorialmanager.com/pone/ and select the 'Submissions Needing Revision' folder to locate your manuscript file.

Please include the following items when submitting your revised manuscript:

- A letter that responds to each point raised by the academic editor and reviewer(s). You should upload this letter as a separate file labeled 'Response to Reviewers'.
- A marked-up copy of your manuscript that highlights changes made to the original version. You should upload this as a separate file labeled 'Revised Manuscript with Track Changes'.
- An unmarked version of your revised paper without tracked changes. You should upload this as a separate file labeled 'Manuscript'.

If you would like to make changes to your financial disclosure, please include your updated statement in your cover letter. Guidelines for resubmitting your figure files are available below the reviewer comments at the end of this letter.

If applicable, we recommend that you deposit your laboratory protocols in protocols.io to enhance the reproducibility of your results. Protocols.io assigns your protocol its own identifier (DOI) so that it can be cited independently in the future. For instructions see: https://journals.plos.org/plosone/s/submission-guidelines#loc-laboratory-protocols. Additionally, PLOS ONE offers an option for publishing peer-reviewed Lab Protocol articles, which describe protocols hosted on protocols.io. Read more information on sharing protocols at https://plos.org/protocols?utm_medium=editorial-email&utm_source=authorletters&utm_campaign=protocols.

We look forward to receiving your revised manuscript.

Kind regards,

Wesley D. Colombo

Academic Editor

PLOS One

Journal Requirements:

If the reviewer comments include a recommendation to cite specific previously published works, please review and evaluate these publications to determine whether they are relevant and should be cited. There is no requirement to cite these works unless the editor has indicated otherwise.

[Note: HTML markup is below. Please do not edit.]

Reviewers' comments:

Reviewer's Responses to Questions

**Comments to the Author**

1. If the authors have adequately addressed your comments raised in a previous round of review and you feel that this manuscript is now acceptable for publication, you may indicate that here to bypass the “Comments to the Author” section, enter your conflict of interest statement in the “Confidential to Editor” section, and submit your "Accept" recommendation.

Reviewer #1: (No Response)

Reviewer #2: All comments have been addressed

**********

2. Is the manuscript technically sound, and do the data support the conclusions?

The manuscript must describe a technically sound piece of scientific research with data that supports the conclusions. Experiments must have been conducted rigorously, with appropriate controls, replication, and sample sizes. The conclusions must be drawn appropriately based on the data presented.

Reviewer #1: No

Reviewer #2: Yes

**********

3. Has the statistical analysis been performed appropriately and rigorously?

Reviewer #1: N/A

Reviewer #2: N/A

**********

4. Have the authors made all data underlying the findings in their manuscript fully available?

The PLOS Data policy requires authors to make all data underlying the findings described in their manuscript fully available without restriction, with rare exception (please refer to the Data Availability Statement in the manuscript PDF file). The data should be provided as part of the manuscript or its supporting information, or deposited to a public repository. For example, in addition to summary statistics, the data points behind means, medians and variance measures should be available. If there are restrictions on publicly sharing data—e.g. participant privacy or use of data from a third party—those must be specified.

Reviewer #1: No

Reviewer #2: Yes

**********

5. Is the manuscript presented in an intelligible fashion and written in standard English?

PLOS ONE does not copyedit accepted manuscripts, so the language in submitted articles must be clear, correct, and unambiguous. Any typographical or grammatical errors should be corrected at revision, so please note any specific errors here.

Reviewer #1: Yes

Reviewer #2: Yes

**********

6. Review Comments to the Author

Please use the space provided to explain your answers to the questions above. You may also include additional comments for the author, including concerns about dual publication, research ethics, or publication ethics. (Please upload your review as an attachment if it exceeds 20,000 characters)

Reviewer #1: Authors Comments:

#3 I would not consider 80 degrees C as soaking, 20-25 C is room temperature 100 c is boiling 80 is much closer to boiling than warm water. Changing the word boiling to soaking is deceptive.

Line notes relate to tracked changes version:

Line 28 It might just be the version I am seeing, but italicize the Latin name, remove larvae from the parentheses.

Line 34 typo on grams

Line 68 the citation added does not back up this claim, this is another improper citation

Line 82 the Latin name needs to stay here, this is the first mention of superworms (after the abstract) but take larvae out of the parenthesis

Line 87 “superworms do not pupate under crowded conditions” citation?

Line 95 its not really samples, it's maybe species or sizes of species. In its current updated form, this sentence doesn’t really tell me anything

Line 106 says the same thing as line 81-83. The effectiveness of superworms was evaluated using a diverse range…

Line 110-113 this is a confusing sentence and it doesn’t say anything but a test was conducted, what were the results? It also doesn’t really fit into this section.

Line 118 replace samples with specimens or animals

Line 123-125 this sentence is confusing and what does the (20) mean?

Line 132 citation

Line 133 I like how you address the longer term health of the colony and provide a solution but line 32-33 make it sound like this may not be a great sustainable alternative to dermestid beetles. On the other had it may be a benefit because you could just feed them food scraps between specimens.

Line 137 3.5 kilograms of larva each month seems like the wrong number, 1000 adult superworms would weigh 450g - 1.36kg, how are they producing 3.5kg of larva each month?

Line 142 check this sentence I think a word is missing but hard to tell with the tracked changes on.

Lines 159, 163, 166 these are the first mentions of these species, add the Latin names, also take out e.g, that is not an example those are the exact and only species you used.

Lines 160 and 161 are contradictory, also was the mummified bat skinned before it was mummified, after, or not at all and did it happen before or after soaking it?

Line 165 did you have a thermometer in the water to confirm? Was the water kept at the same temperature for 20-30 minutes or was it heated and then allowed to cool. Fresh tissue is already soft so I still don’t understand why this step was necessary, especially with the potential damage it could cause to the bones. How did you choose 20-30 minutes? Did you see a clear change in the tissue after that process? Was it falling off the bones?

Line 168 how many containers were used for the large specimens? What is the value of using large specimens if you separate them out into multiple containers that might be comparable to the medium specimens

Line 169 based on the larva? Fix the tense used

Line 171, 174 specimen not sample

For table 1 to be useful you also need to capture the clean weight of the specimen to determine how much tissue was eaten/removed, which would tell you what the consumption rate was, and you could use that formula for any sized specimen. What is the explanation for why the alligator gar took 10 more hours than the Eurasian eagle hour when they had similar ratios? Make sure you capitalization is consistent. It also needs to be clarified how the times for the large animals in multiple containers worked. Were the containers all started at the same time for an animal or did you wait for one container to finish before starting the next one?

Line 188 how do you know 700 grams enhances cleaning efficiency?

Line 189 this adds a new variable that was not previously mentioned and runs counter to having 700 grams of larva if you are going to replace them, why not just have more larvae? Or did you mean to say within instead of between? The gray wolf was rotated every 6 hours for 78 hours? How was this time period determined?

Line 128, 194 these say the same thing

Line 189, 195 why 6-7 hours and 6-8 hours? How do you know the Egyptian Rousette was done in one hour if it was monitored every 6-8 hours?

Fig 2 why did you use a picture of a species not included in this study? It also looks like B the bird has been turned over and there are significantly fewer larvae, were the larvae replaced after 6 hours?

Line 203 the maintenance for superworms seems to be higher than dermestid beetles, as a tip try putting a wet paper towel over the specimen, it will slow down the drying process

Line 208 this is repetitive

Line 215 no explanation of why the bleach solution was used and bleach can damage bones.

Line 222 if you tried those other methods it seems like you should have tried dermestids, which would be the closest comparison to superworms

Line 235,6 you already established the size parameters, you can remove the entire parentheses sections here

Line 237-239 and 248-251 say the same thing and are both also shown in the table not to mention being an obvious result, more mass takes longer.

Line 257 I dn’t think you can make this claim when none of the ratios you used fell between 10-15 your results do not clearly support this.

Line 259 You just introduced an entirely new set of specimens that were not mentioned anywhere above, and they are not comparable because you only used skulls, not the entire skeletons, how would you know if it worked for the rib bones without damage? You are also comparing damage to two mammals to birds, why did you not use mammals as a comparison? This table should also include larval biomass. In multiple places you state that 700 grams is the preferred amount of larvae but to get those ratios you would have needed to use less than 700 grams negating your claims that 700 grams is optimal. The cleaning time also significantly increased.

Line 268 where in your results can you show me that soaking accelerated the process? You would need a control to make this claim and you do not have one. Also if the tissue was so softened why did it need to get re-soaked every 12 hours?

Line 269 what internal areas? You already removed all the skin, excess tissue and organs

Line 268-271 you said the same thing twice

Line 275 fig 4 is not a close enough picture to see any type of damage to the bones, especially if they have already been covered in a varnish

Line 291 what facilities are using chemical treatments?

Line 306 local to where? This is an international publication your local and your readers local are likely not the same

Line 315 your citation number is wrong (unless it’s a tracked changes issue)

Line 318-9 you say they don’t fly then you say the can fly, also missing a citation

Line 345/table 2 none of the animals listed in that table are the same as the animals you used. There is no indication of how those specimens were prepared, they could have been complete animals with skin/fur/organs still present. No indication of how many dermestids were used. You do not have the evidence to support your claim that “superworms demonstrated a markedly shorter cleaning duration compared to dermestid beetles.”

Table 2 citations appear to be wrong for cat, roe deer, cow

Line 347 saying “beetle-based cleaning often requires several weeks is deceptive, the human jawbone is clearly an outlier and the only other one was an entire deer which is much larger than your large category. Your “large” is not the same as large, a clean cow skull by itself could weigh 3-4kg.

Line 357 not supported by your results

Line 360 700 grams of larvae were not used for your extra owl skulls.

Line 364-5 you are doing things that you can’t make a clear comparison to dermestid beetles, did any of the specimens listed in table 2 use starving dermestids or switch them out every 6-8 hours?

Line 366 again, you do not have a control that proves that repositioning made a difference

Line 372 this is the first mention of odor

Line 375 missing citation, who recommends?

Line 377 Citation number is wrong

Reviewer #2: I believe that this revision of the manuscript is more comprehensive in terms of its methods and intentions.

The cleaning of osteological material has been the subject of various studies, so having an alternative to chemical methods and dermestid beetle cultures is extremely helpful, especially in countries where it is difficult to maintain laboratories with the appropriate conditions for handling chemicals or where dermestid beetles are difficult to obtain.

The publication of these results in this journal is important given its international reach, offering an alternative or coexistence with other chemical or biological methods of cleaning bone material.

**********

7. PLOS authors have the option to publish the peer review history of their article (what does this mean?). If published, this will include your full peer review and any attached files.

If you choose “no”, your identity will remain anonymous but your review may still be made public.

**Do you want your identity to be public for this peer review?** For information about this choice, including consent withdrawal, please see our Privacy Policy.

Reviewer #1: No

Reviewer #2: No

**********

[NOTE: If reviewer comments were submitted as an attachment file, they will be attached to this email and accessible via the submission site. Please log into your account, locate the manuscript record, and check for the action link "View Attachments". If this link does not appear, there are no attachment files.]

To ensure your figures meet our technical requirements, please review our figure guidelines: https://journals.plos.org/plosone/s/figures

You may also use PLOS’s free figure tool, NAAS, to help you prepare publication quality figures: https://journals.plos.org/plosone/s/figures#loc-tools-for-figure-preparation.

NAAS will assess whether your figures meet our technical requirements by comparing each figure against our figure specifications.

# Author comment: Author response to Decision Letter 2

**DOI:** 10.1371/journal.pone.0349669.r004

14 Apr 2026

Dear Editor, dear Reviewers,

We are pleased to submit the revised version of our manuscript, “A practical and safe alternative method for skeletal cleaning for museum specimens using superworms (Zophobas morio).” We sincerely thank the Editor and Reviewers for their thoughtful and constructive feedback. We have carefully addressed every comment and point raised, as detailed below. Notably, we have revised our terminology, an issue which was central to concerns with the original submission, and provided additional experimental details to clarify our methodology and improve reproducibility. We believe these major revisions address and resolve the key points raised, including those highlighted by academic editor and reviewer 1. We hope that the Editor and Reviewers find our revised manuscript suitable for publication in PLOS ONE.

With kind regards on behalf of the authors,

Morteza Monfared

RESPONSES TO AE AND REVIEWER COMMENTS

The line numbers referenced in our responses correspond to the clean version of the manuscript without tracked changes.

Academic editors

Editors’ comments to the author:

Thank you for submitting your manuscript to PLOS ONE. After careful consideration, we feel that it has merit but does not fully meet PLOS ONE’s publication criteria as it currently stands. Therefore, we invite you to submit a revised version of the manuscript that addresses the points raised during the review process. Based on Reviewer 1’s comments, the manuscript requires substantial revision before it can be reconsidered for publication. The review identifies significant shortcomings in methodological rigor, clarity, and evidentiary support, which currently undermine several of the manuscript’s main conclusions.

A central concern is the use of misleading terminology. Referring to water at 80 °C as “soaking” is inappropriate and must be corrected. The procedure should be described precisely, including how temperature was measured, whether it was maintained or allowed to cool, and the exact duration of exposure.

Several key conclusions are not supported by the experimental design. Claims that soaking accelerated cleaning or that repositioning specimens improved efficiency lack proper controls and therefore must be removed or clearly rephrased as descriptive observations rather than causal statements.

The comparison with dermestid beetles is also problematic. The manuscript does not provide comparable data to support claims of superior cleaning speed, and Table 2 includes inconsistent specimens and questionable citations. This comparison should be removed, substantially revised, or strongly qualified.

Methodological details are insufficient in multiple areas, including larval replacement, specimen repositioning, number of containers used for large specimens, monitoring intervals, and criteria for determining completion. These variables must be clearly defined to ensure reproducibility.

In addition, several numerical claims, particularly regarding the optimal use of 700 g of larvae and monthly larval production, are not adequately supported and appear inconsistent. These statements require justification or removal.

The manuscript also contains redundancy, unclear statements, inconsistent terminology (e.g., “samples” vs. “specimens”), and errors in scientific naming and citation. Tables and figures need revision to provide meaningful quantitative information and visual evidence that supports the claims made.c

In summary, major revision is required. The authors must address methodological gaps, remove or qualify unsupported claims, improve clarity and consistency, and correct all issues related to data presentation and citations before the manuscript can be reconsidered for publication.

Authors’ Response: We sincerely thank the Academic Editor for the careful consideration and feedback on our manuscript. We have thoroughly revised the manuscript to address all major concerns and bring the work in line with PLOS ONE’s publication criteria.

Misleading terminology

We acknowledge that referring to the pre-cleaning step as “soaking” at 80 °C was imprecise and potentially misleading. The reason we changed the term from "boiling" to "soaking" was that our process did not involve a true boil; however, we appreciate that "soaking" does not accurately reflect the conditions either. In the revised manuscript, we have completely rephrased this process to describe it as “softening tissues in hot water at 80 °C.” We now provide a precise methodological description, explicitly noting how temperature was measured and maintained, and the exact duration of exposure for each specimen type (please see methods, line 164-169 and 175-176).

Unsupported claims and experimental control

We have carefully reviewed all conclusions and have removed or rephrased any claims that were not directly supported by our experimental design or the available controls. For example, regarding the pre-treatment step and its impact on cleaning efficiency, we now provide a detailed explanation in the text of how the experiment was set up and specifically why this step is suggested to accelerate the process. For the tissue-softening step, we used a laboratory heater with a thermometer to maintain the water temperature at 80 °C for 20–30 minutes, with duration determined by preliminary trials to ensure effective softening and no bone damage. These specifics have been clarified in the text (please see method, line164-169). To evaluate the effect of this pre-treatment, we conducted a comparative trial with two wolf skulls of similar size: one underwent softening at 80 °C before larval exposure, the other did not. The pre-treated skull had about a 50% reduction in cleaning time and showed no bone damage (please see also our detailed response to Reviewer 1, comment 19").

Comparison with dermestid beetles

We totally agree that we cannot directly compare our results with those from the studies listed in Table 2 due to inconsistencies in specimen type and methodological details. The specimens referenced, which were cleaned by dermestid beetles, are drawn from various studies and do not directly match the species, specimen preparation, or cleaning protocols used in our work. As you and reviewer 1 noted, important methodological details such as dermestid pre-conditioning (e.g., starving, regular replacement) are not stated, making the cleaning durations difficult to compare. Therefore, all comparative claims of superior cleaning speed by superworms have been removed. We now in this revised version of manuscript only discussed the result that we got from our experiment.

Methodological clarity and numerical claims

We have expanded the Methods section to clearly define all important variables, we explained our setup and the limitation and the process that we did the experiment with details. We try in this version clearly define all procedures and variables, including larval replacement, specimen handling, rotation, monitoring intervals, use of containers, and completion criteria (please see the first part of method section and also in the response to the reviewer 1 comments 20 and 23, 24).

Clarity and consistency

The manuscript has been thoroughly edited to remove redundancy, clarify ambiguous statements, standardize terminology, and correct all errors in scientific names and citations. Tables and figures have been revised and now provide quantitative information and clear visual documentation directly supporting our claims.

Reviewer 1

#1 - I would not consider 80 degrees C as soaking, 20-25 C is room temperature 100 c is boiling 80 is much closer to boiling than warm water. Changing the word boiling to soaking is deceptive.

Authors’ Response: Thank you for your comment and for highlighting issues with our terminology. We apologize for any confusion caused by our initial description. The reason we changed the term from "boiling" to "soaking" was that our process did not involve a true boil; however, we appreciate that "soaking" does not accurately reflect the conditions either. To clarify, our methodology involved softening the tissue by placing the specimens in water maintained at 80°C, using a laboratory heater and thermometer. Once the water reached 80°C, the specimens were placed in the water, and we kept the temperature as close to 80°C as possible throughout the soften process. This process was selected to soften muscle and connective tissues and to help remove fats, thereby improving the efficiency of subsequent larval cleaning. Based on your remarks, we acknowledge that "soaking" can be misleading and that "boiling" is not accurate for this process. In the revised manuscript, we now called this step as "softening tissue process in hot water at 80°C". We believe this terminology accurately reflects our procedure and provides clarity for readers.

#2 - Line 28 It might just be the version I am seeing, but italicize the Latin name, remove larvae from the parentheses.

Authors’ Response: It’s done.

#3 - Line 34 typo on grams

Authors’ Response: It’s done.

#4 - Line 68 the citation added does not back up this claim, this is another improper citation

Authors’ Response: The better citation is added.

#5 - Line 82 the Latin name needs to stay here, this is the first mention of superworms (after the abstract) but take larvae out of the parenthesis

Authors’ Response: It’s done.

#6 - Line 87 “superworms do not pupate under crowded conditions” citation?

Authors’ Response: It’s done.

#7 - Line 95 its not really samples, it's maybe species or sizes of species. In its current updated form, this sentence doesn’t really tell me anything

Authors’ Response: You are right, and in this new revised version this sentence is removed from the manuscript.

#8 - Line 106 says the same thing as lines 81-83. The effectiveness of superworms was evaluated using a diverse range….

Authors’ Response: We have removed the redundant statement from the beginning of the Methods section.

#9 - Line 110-113 this is a confusing sentence and it doesn’t say anything but a test was conducted, what were the results? It also doesn’t really fit into this section.

Authors’ Response: Our intention in this section was to introduce a comparative trial between a traditional non-biological method (boiling in hot water) and the superworm cleaning approach, in order to highlight the differences in risk and processing time. We have reworded this part of the Methods section to state the comparative aim more explicitly, and we have clearly indicated that the corresponding results—regarding risk of specimen damage—are presented in the Results section (see Line 99-102 in method and line 222-226 in Result). If you feel this information disrupts the methodological flow, we are also open to moving this description to the Results or Discussion section for improved clarity and readability.

#10 - Line 118 replace samples with specimens or animals

Authors’ Response: It’s done.

#11 - Line 123-125 this sentence is confusing and what does the (20) mean?

Authors’ Response: This sentence has been rewritten in the revised manuscript for greater clarity.

#12 - Line 132 citation

Authors’ Response: It’s done.

#13 - Line 133 I like how you address the longer term health of the colony and provide a solution but

#14 - line 32-33 make it sound like this may not be a great sustainable alternative to dermestid beetles. On the other had it may be a benefit because you could just feed them food scraps between specimens.

Authors’ Response: Thank you for your comment. We have added this point, with the reference, to the manuscript (see Lines 123-129). Additionally, we have revised all sections where we compare the maintenance of dermestid beetles with superworms to ensure clarity and accuracy throughout the text.

#15 - Line 137 3.5 kilograms of larva each month seems like the wrong number, 1000 adult superworms would weigh 450g - 1.36kg, how are they producing 3.5kg of larva each month?

Authors’ Response: The weight of adult superworms does not directly correspond to the total larval biomass produced, as adults can reproduce multiple times each month under optimal conditions. Each female is capable of laying dozens of eggs per month, and with proper husbandry, a significant percentage of these eggs develop into larvae. We have been successfully maintaining and operating our superworm colony for several years, consistently producing up to 3.5 kilograms of larvae each month under suitable conditions.

#16 - Line 142 check this sentence. I think a word is missing but hard to tell with the tracked changes on.

Authors’ Response: It’s corrected.

#17 - Lines 159, 163, 166 these are the first mentions of these species, add the Latin names, also take out e.g, that is not an example those are the exact and only species you used.

Authors’ Response: It’s added.

#18 - Lines 160 and 161 are contradictory, also was the mummified bat skinned before it was mummified, after, or not at all and did it happen before or after soaking it?

Authors’ Response: The bat specimen was already dried and mummified prior to any preparation in our study, and it was not skinned either before or after mummification. To soften the tissues and facilitate larval cleaning, we placed the intact mummified specimen in hot water at up to 80°C for 5 minutes before placing it in the larval container. This procedure has been clarified in the revised manuscript (see lines 158-161).

#19 - Line 165 did you have a thermometer in the water to confirm? Was the water kept at the same temperature for 20-30 minutes or was it heated and then allowed to cool. Fresh tissue is already soft so I still don’t understand why this step was necessary, especially with the potential damage it could cause to the bones. How did you choose 20-30 minutes? Did you see a clear change in the tissue after that process? Was it falling off the bones?

Authors’ Response: Yes, we used a laboratory heater with a thermometer throughout the process to ensure that the water temperature was maintained as close to 80°C as possible for the entire 20–30 minutes period. The soften duration was determined through preliminary trials, during which we tested various time intervals and evaluated each sample for effective tissue softening and potential bone damage. We consistently found that the 20–30 minutes range was both safe and effective across different specimens, with the precise duration adjusted according to the size and maturity of each sample. We make it clearer in the text also (see lines 164-169). To assess the necessity and impact of this step, we conducted a comparative trial using two wolf skulls of similar size and weight: for one skull we used the soften process at 80°C prior to larval exposure, while the other was not. The skull subjected to the pretreatment process exhibited approximately a 50% reduction in cleaning time, with no observable bone damage or excessive tissue detachment. Based on these results, we included this pre-treatment step to significantly enhance the overall efficiency of the cleaning process without compromising specimen integrity.

#20 - Line 168 how many containers were used for the large specimens? What is the value of using large specimens if you separate them out into multiple containers that might be comparable to the medium specimens

Authors’ Response: Due to the use of fixed-size containers (each measuring 40 cm × 30 cm × 12 cm) in our experimental design, large specimens could not be accommodated in a single container. As a result, each large specimen was divided into multiple containers, with each container receiving 700 grams of larvae. This amount of larvae represents the maximum larval density that allows for optimal humidity, temperature, and larval activity within one container (just based on the size of containers that we use in our experiments). For clarity, we have added this explanation to the manuscript (see lines 177-185 and in discussion in part “Optimizing cleaning efficiency”). For example, the Gray Wolf was divided among four containers and the Wild Cat among three containers. In our trials, attempting to fit more larvae into a single container led to overcrowding activity (please see reference 20), which reduced cleaning effic

Attachment Submitted filename: Response-to-Reviewers.docx

# Peer review: Decision Letter 2

**DOI:** 10.1371/journal.pone.0349669.r005

**Authors:** Wesley D. Colombo

4 May 2026

A practical and safe alternative method for skeletal cleaning for museum specimens using superworms (Zophobas morio)

PONE-D-25-57203R2

Dear Dr. Monfared,

We’re pleased to inform you that your manuscript has been judged scientifically suitable for publication and will be formally accepted for publication once it meets all outstanding technical requirements.

Within one week, you’ll receive an e-mail detailing the required amendments. When these have been addressed, you’ll receive a formal acceptance letter and your manuscript will be scheduled for publication.

An invoice will be generated when your article is formally accepted. Please note, if your institution has a publishing partnership with PLOS and your article meets the relevant criteria, all or part of your publication costs will be covered. Please make sure your user information is up-to-date by logging into Editorial Manager at Editorial Manager® and clicking the ‘Update My Information' link at the top of the page. For questions related to billing, please contact billing support.

If your institution or institutions have a press office, please notify them about your upcoming paper to help maximize its impact. If they’ll be preparing press materials, please inform our press team as soon as possible -- no later than 48 hours after receiving the formal acceptance. Your manuscript will remain under strict press embargo until 2 pm Eastern Time on the date of publication. For more information, please contact onepress@plos.org.

Kind regards,

Wesley D. Colombo

Academic Editor

PLOS One

Additional Editor Comments (optional):

Reviewers' comments:

Reviewer's Responses to Questions

**Comments to the Author**

1. If the authors have adequately addressed your comments raised in a previous round of review and you feel that this manuscript is now acceptable for publication, you may indicate that here to bypass the “Comments to the Author” section, enter your conflict of interest statement in the “Confidential to Editor” section, and submit your "Accept" recommendation.

Reviewer #1: (No Response)

**********

2. Is the manuscript technically sound, and do the data support the conclusions?

The manuscript must describe a technically sound piece of scientific research with data that supports the conclusions. Experiments must have been conducted rigorously, with appropriate controls, replication, and sample sizes. The conclusions must be drawn appropriately based on the data presented.

Reviewer #1: Yes

**********

3. Has the statistical analysis been performed appropriately and rigorously?

Reviewer #1: N/A

**********

4. Have the authors made all data underlying the findings in their manuscript fully available?

The PLOS Data policy requires authors to make all data underlying the findings described in their manuscript fully available without restriction, with rare exception (please refer to the Data Availability Statement in the manuscript PDF file). The data should be provided as part of the manuscript or its supporting information, or deposited to a public repository. For example, in addition to summary statistics, the data points behind means, medians and variance measures should be available. If there are restrictions on publicly sharing data—e.g. participant privacy or use of data from a third party—those must be specified.

Reviewer #1: Yes

**********

5. Is the manuscript presented in an intelligible fashion and written in standard English?

PLOS ONE does not copyedit accepted manuscripts, so the language in submitted articles must be clear, correct, and unambiguous. Any typographical or grammatical errors should be corrected at revision, so please note any specific errors here.

Reviewer #1: Yes

**********

6. Review Comments to the Author

Please use the space provided to explain your answers to the questions above. You may also include additional comments for the author, including concerns about dual publication, research ethics, or publication ethics. (Please upload your review as an attachment if it exceeds 20,000 characters)

Reviewer #1: Author comments based on tracked changes version:

Line 111 specimens not samples

Line 132 (14) should be [14]

Line 159, 166, 177 are missing the closing parentheses for all three

line 215 why is the hooded crow not listed in table 1, was this specimen part of the study or not, this seems to be its only mention in the manuscript. If this stays it would also need the Latin name.

**********

7. PLOS authors have the option to publish the peer review history of their article (what does this mean?). If published, this will include your full peer review and any attached files.

If you choose “no”, your identity will remain anonymous but your review may still be made public.

**Do you want your identity to be public for this peer review?** For information about this choice, including consent withdrawal, please see our Privacy Policy.

Reviewer #1: No

**********

# Editor report: Acceptance letter

**DOI:** 10.1371/journal.pone.0349669.r006

**Authors:** Wesley D. Colombo

PONE-D-25-57203R2

PLOS One

Dear Dr. Monfared,

I'm pleased to inform you that your manuscript has been deemed suitable for publication in PLOS One. Congratulations! Your manuscript is now being handed over to our production team.

At this stage, our production department will prepare your paper for publication. This includes ensuring the following:

* All references, tables, and figures are properly cited

* All relevant supporting information is included in the manuscript submission,

* There are no issues that prevent the paper from being properly typeset

You will receive further instructions from the production team, including instructions on how to review your proof when it is ready. Please keep in mind that we are working through a large volume of accepted articles, so please give us a few days to review your paper and let you know the next and final steps.

Lastly, if your institution or institutions have a press office, please let them know about your upcoming paper now to help maximize its impact. If they'll be preparing press materials, please inform our press team within the next 48 hours. Your manuscript will remain under strict press embargo until 2 pm Eastern Time on the date of publication. For more information, please contact onepress@plos.org.

You will receive an invoice from PLOS for your publication fee after your manuscript has reached the completed accept phase. If you receive an email requesting payment before acceptance or for any other service, this may be a phishing scheme. Learn how to identify phishing emails and protect your accounts at https://explore.plos.org/phishing.

If we can help with anything else, please email us at customercare@plos.org.

Thank you for submitting your work to PLOS ONE and supporting open access.

Kind regards,

PLOS ONE Editorial Office Staff

on behalf of

Dr. Wesley D. Colombo

Academic Editor

PLOS One
